# Simulation of Automatically Annotated Visible and Multi-/Hyperspectral Images Using the Helios 3D Plant and Radiative Transfer Modeling Framework

**DOI:** 10.34133/plantphenomics.0189

**Published:** 2024-05-30

**Authors:** Tong Lei, Jan Graefe, Ismael K. Mayanja, Mason Earles, Brian N. Bailey

**Affiliations:** ^1^ Department of Plant Sciences, University of California, Davis, CA, USA.; ^2^ Leibniz Institute of Vegetable and Ornamental Crops e.V. (IGZ), Großbeeren, Germany.; ^3^ Department of Biological and Agricultural Engineering, University of California, Davis, CA, USA.; ^4^ Department of Viticulture and Enology, University of California, Davis, CA, USA.

## Abstract

Deep learning and multimodal remote and proximal sensing are widely used for analyzing plant and crop traits, but many of these deep learning models are supervised and necessitate reference datasets with image annotations. Acquiring these datasets often demands experiments that are both labor-intensive and time-consuming. Furthermore, extracting traits from remote sensing data beyond simple geometric features remains a challenge. To address these challenges, we proposed a radiative transfer modeling framework based on the Helios 3-dimensional (3D) plant modeling software designed for plant remote and proximal sensing image simulation. The framework has the capability to simulate RGB, multi-/hyperspectral, thermal, and depth cameras, and produce associated plant images with fully resolved reference labels such as plant physical traits, leaf chemical concentrations, and leaf physiological traits. Helios offers a simulated environment that enables generation of 3D geometric models of plants and soil with random variation, and specification or simulation of their properties and function. This approach differs from traditional computer graphics rendering by explicitly modeling radiation transfer physics, which provides a critical link to underlying plant biophysical processes. Results indicate that the framework is capable of generating high-quality, labeled synthetic plant images under given lighting scenarios, which can lessen or remove the need for manually collected and annotated data. Two example applications are presented that demonstrate the feasibility of using the model to enable unsupervised learning by training deep learning models exclusively with simulated images and performing prediction tasks using real images.

## Introduction

Remote and proximal sensing of plant systems enables non-intrusive monitoring of plant architecture, composition, and biophysical state with high-throughput [1–4]. Advances in modern remote and proximal sensing technology have resulted in an abundance of high-resolution images and sensor data of natural and managed vegetation systems, which have the potential to provide comprehensive insights into plant function, to accelerate and broaden modern breeding pipelines, and to provide actionable information to managers [4–6]. Multi- and hyperspectral imaging has emerged as a promising sensing mode, as it can quantify plant characteristics not visible to the eye by detecting interactions between radiation and plant tissues [3]. Analyzing spectral signatures of reflected radiation contained in images has enabled applications in plant high-throughput phenotyping and horticultural management such as citrus greening detection [7], measurement of canopy structure, and biochemical properties of crops [8], assessing leaf traits including chlorophyll, water, dry matter, and nitrogen content [1,2], yield estimation [9,10], and interactive effects of water and nitrogen in irrigated horticultural crop production [6], among many others. Visible imaging is a much more accessible but limited optical sensing technique that can be regarded as a form of multispectral imaging with only 3 bands in the visible region of the electromagnetic spectrum. It is most useful for detection tasks commonly done visually by humans such as plant counting, growth monitoring, and the identification of disease symptoms [2,11], but has limited capability for quantifying biochemical properties or physiological processes. Many previous studies have combined visible with multispectral [8,10], thermal [12], or depth imaging [13–15] for high-throughput plant phenotyping.

Despite the promise of remotely sensed data to enable reliable, high-throughput measurement of plant traits and function, linking remote and proximal sensing imagery to plant traits useful for decision-making has remained a challenge. At its core, remote sensing is a radiative transfer problem, which has led to the development of an incredibly wide array of radiative transfer models aimed at better interpreting remote sensing data [16,17]. Early models were simple enough that they could accept remote sensing data as input and be directly inverted for plant traits of interest [16,18]. Models have evolved to become highly complex such that they can fully resolve vegetation geometry in 3 dimensions and represent relevant modes of radiation transfer across its spectrum [17,19,20]. However, their complexity makes them difficult to invert based on remote sensing data inputs, and these methods are constrained by the absence of direct connections to plant biophysical processes, which play a crucial role in determining how photons interact with plant tissues.

In the absence of detailed, physically based models to facilitate automated trait extraction from remotely sensed imagery, a popular alternative has been the use of computer vision techniques, which have been revolutionized in recent years by rapid advances in machine learning [5,7]. However, the most widely used machine learning models, including deep learning models, are supervised and require an exceptionally large amount of high quality and often manually annotated data for model training, which necessitates expert knowledge and remains tedious and time-consuming (or impossible depending on the trait). For instance, image annotation for wheat spikelet and ear counting is typically done manually [21], and manual selection of the canopy region of interest (ROI) is also required for many methods [7]. Additionally, the fusion of multimodal data also faces challenges when aligning images captured by different sensors due to their physically distinct viewpoints and resolutions [14,15].

Although several publicly available annotated image datasets for agricultural applications are available for machine learning model training and other plant phenotyping applications, such as the Annotated Crop Image Dataset [21], Michigan State University Plant Imagery Dataset (MSU-PID) [13], AgML [22], and KOMATSUNA dataset [15], these data repositories are not sufficiently broad to capture the extensive variability that exists within agricultural machine learning tasks. The limitations of machine learning approaches become evident with small and low-variation datasets, as they can lead to severe overfitting, and the resulting models are often not readily transferable across different light conditions, species, or phenotyping platforms, revealing a lack of generalization and posing a substantial risk of extrapolation errors [2,22]. Past researchers have utilized data augmentation methods like random cropping, scaling, rotation, and flipping in the spatial domain [21,23], and introduction of random variations in mean offset and slope of the spectral reflectance [24]. However, in many cases, these methods insufficiently describe the variation of sample distributions caused by changes in plant species, lighting conditions, or sensors.

**Fig. 1.**
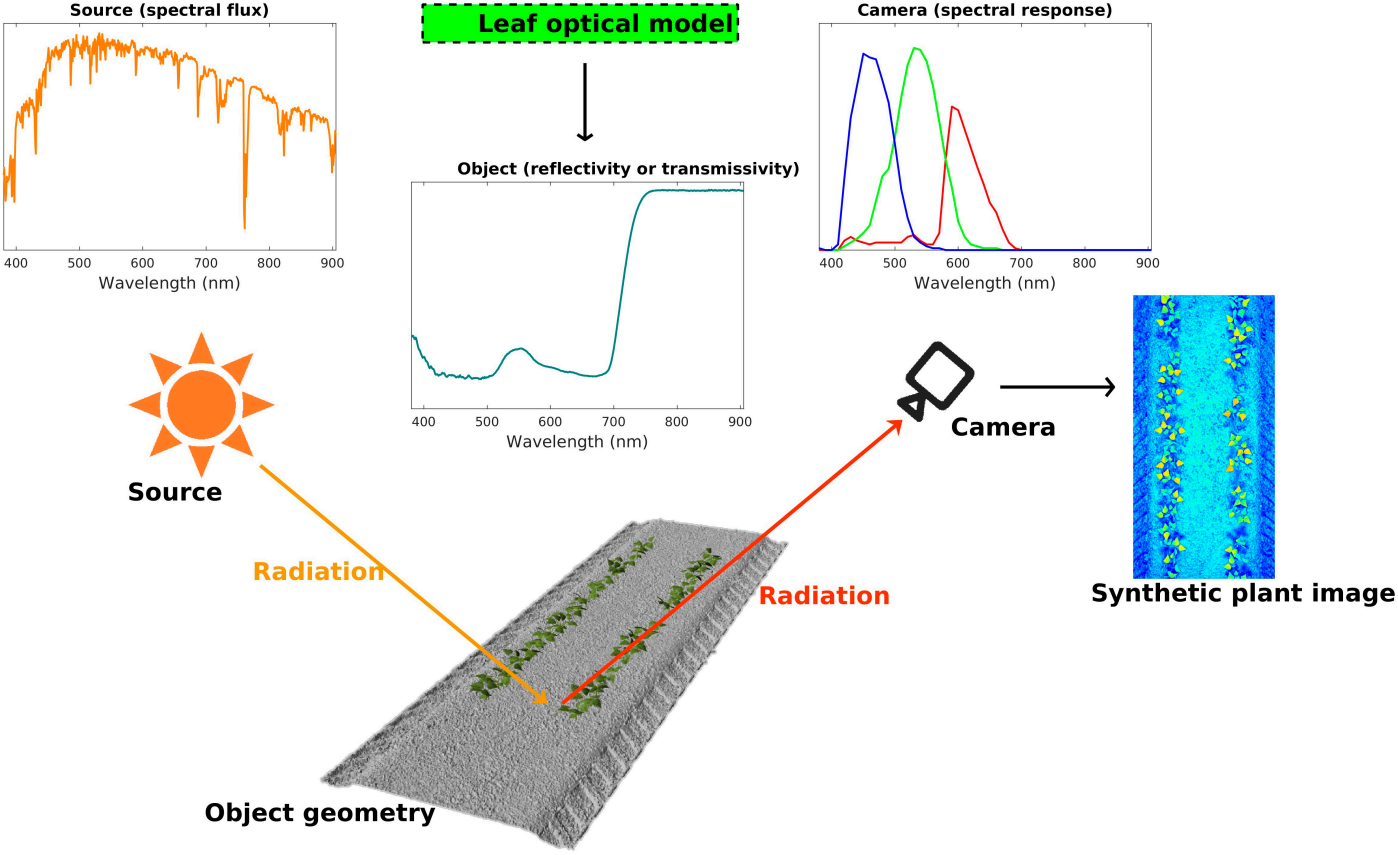
Schematic representation of the synthetic imagery generation framework. A ray-tracing-based camera model is used to simulate radiation propagation that is emitted from a source (e.g., sun and LED light) and reaches the camera after being scattered by objects in the scene. Spectral distributions of radiation source fluxes, object reflectivity and transmissivity, and camera response are specified to define how radiation interacts with object surfaces and the camera sensor. The PROSPECT-based leaf optical model can generate the leaf optical properties according to the user-specified chemical concentrations of leaves. Finally, the simulated camera generates resulting images that can be arbitrarily auto-annotated.

This work presents a novel 3-dimensional (3D) radiative transfer modeling framework for simulation of visible, multi-/hyperspectral, depth, and thermographic imagery that can be readily coupled with machine learning models for inversion based on flexible, automated image annotation (Fig. 1). This allows the machine learning model to effectively serve as an inverter of the 3D model. The radiation model is an extension of the Helios 3D plant modeling software [25], which enables direct coupling of the radiative transfer simulations with the biophysical simulation capabilities of Helios, such as photosynthesis, transpiration, energy transfer, etc. The automated annotation capability can mitigate the high cost of obtaining large datasets for training machine learning models, enable a wide parameter space to be incorporated within machine learning model training, and enable high-throughput phenotyping of traits that may be impossible to measure or “annotate” at scale [26]. In comparison to other radiative transfer-based image synthesis models, such as the LargE-Scale remote sensing data and image simulation framework (LESS) [20] and the Discrete Anisotropic Radiative Transfer (DART)-Lux model [27], the current framework can perform simulations of radiation transport on both large and small scales for plants while providing both geometric and biophysical annotations. The main components of the present image synthesis framework consist of 4 modules:

1. Ray-tracing model: a graphics processing unit (GPU)-accelerated “reverse” ray-tracing method [19] computes the distribution of absorbed, transmitted, reflected, and emitted radiation for all geometric objects in the domain based on light sources with arbitrary spectral flux distribution. A camera sensor is simulated by calculating the radiative flux recorded by each camera pixel based on its intrinsic and extrinsic properties, which produces final synthetic images.

2. Automated image annotation: Every pixel in the simulated images can be automatically annotated based on arbitrary identifiers assigned to geometric elements in the scene, or based on any biophysical variable computed in Helios (e.g., net photosynthetic flux, transpiration flux, stomatal conductance, and chemical compound concentrations).

3. Camera calibration model: a calibration procedure is developed to specify the camera spectral response and recovery of image distortion based on calibration images captured by real-world target cameras.

4. Leaf optics model: The PROSPECT model [28,29] is used to simulate the optical properties of leaf tissues according to the concentration of specified compounds such as chlorophyll, carotenoids, and water, which themselves may be determined by coupling with Helios biophysical models.

In this paper, we present the principles and verification of the above modules. Examples of synthetic plant images along with their corresponding labeling maps are provided, along with 2 case studies examining the utility of incorporating synthetic imagery into machine-learning-based plant phenotyping. The framework has been incorporated into the Radiation plug-in of the Helios modeling software (v1.3.0), available for free download at https://github.com/PlantSimulationLab/Helios. Documentation can be found at https://baileylab.ucdavis.edu/software/helios/.

## Methods

### Model geometry

3D meshes of planar primitive elements form the basis of geometric objects contained within simulated domains (object geometry in Fig. 1). The Helios modeling software provides a means for generation and manipulation of fully resolved 3D geometric models of plants, the ground, or other objects [25]. Plug-ins included within Helios can be used to quickly create procedurally generated models with random variation within a prescribed range for different types of plant geometries such as bean, strawberry, sorghum, and walnut trees, as well as a ground surface made up of multiple textured patches. The leaf geometry is formed using triangular or rectangular primitive elements, which can be masked using the transparency channel of a PNG image file to create planar elements with any desired shape. The procedural models have user-defined geometric parameters such as canopy height, leaf size, leaf area index (LAI), and leaf angle distribution, which allows for easy customization of the specifics of the canopy, and can be used as labels for output images in both large and small scales. Helios can also import external geometries from standard polygon file formats (such as “.ply” and “.obj”). Geometry added to the scene can be referenced based on their unique identifiers in order to assign their spectral radiative properties.

### Ray-tracing model description

#### Overview

Simulated images are generated and labeled by fusing a camera model with the existing radiation transfer model in Helios [19]. In a first pass, the distribution of absorbed, reflected, transmitted, and emitted radiation for all primitive elements in the scene is computed for a single scattering iteration (i.e., single scattering or emission instance) based on the ray-tracing method proposed by Bailey [19]. A ray-tracing-based camera model is then used to sample the reflected and transmitted energy for every camera pixel across all wave bands. Scattering iterations continue for multiple scattering instances, and the camera continues accumulating scattered radiation until the amount of remaining scattered radiation becomes arbitrarily small. The camera also uses ray-tracing to determine primitive elements contained within each pixel, which is then used for image labeling. The reverse ray-tracing approach utilized in this study for emission and scattering ensures adequate ray sampling in the presence of complex geometry with very small and skewed primitive elements [19].

Details on each of these components of the model are given in the sections below.

#### Radiation sources and surface radiative properties

Radiation originates in the scene due to 8 potential sources: (a) collimated solar radiation, (b) radiation emanating from a sphere with the same radius and distance from Earth as the Sun (thus incorporating penumbral effects), (c) diffuse solar radiation with specified angular distribution, (d) a terrestrial spherical source (e.g., a light bulb), (e) a terrestrial disk-shaped source emitting radiation from one side (e.g., halogen lamp), (f) a terrestrial rectangular-shaped source emitting radiation from one side (e.g., an LED array), (g) longwave radiation emitted by terrestrial objects (i.e., primitives), and (h) diffuse longwave radiation emitted by the sky. We classify source types a to f as “external” radiative sources, and types g and h as “longwave” radiative sources. Each radiation source is defined based on its emitted flux integrated across each radiation band considered in the model, its position/orientation, and its spectral distribution (for external sources only). Emission from spherical external sources is isotropic, and emission from planar external sources follows a cosine distribution. The spectral distribution of longwave sources is not considered explicitly, as their fluxes are specified from the integral over all longwave wavelengths based on their temperature according to Stefan–Bolzmann law.

Before running the ray-tracing model, a reflectance (*ρ_λ_*) and transmittance (*τ_λ_*; if applicable) spectrum is assigned to each primitive in the scene. These surface spectra are then integrated over each user-defined spectral band in the standard way [30] to yield the total reflectivity (*ρ*) and transmissivity (*τ*) for each band. These values are used in the calculation of radiative exchange between objects in the scene via scattering as described previously by Bailey [19].

For radiation exchange between objects and the camera, different integrated surface radiative properties are used that account for the spectral sensitivity of the camera sensor. The surface reflectivity *ρ_o_* and transmissivity *τ_o_* used for radiation scattered to the camera sensor is calculated based on the specified surface spectral radiative properties mentioned above, and the camera spectral response for each band as follows:

$$\rho_{o}=\frac{\int_{\lambda_{\min}}^{\lambda_{\max}}\;\rho_{\lambda }\;C_{\lambda }\;S_{\lambda }\;d\lambda }{\int_{\lambda_{\min}}^{\lambda_{\max}}\;S_{\lambda }\;d\lambda },$$ (1)

$$\tau_{o}=\frac{\int_{\lambda_{\min}}^{\lambda_{\max}}\;\tau_{\lambda }\;C_{\lambda }\;S_{\lambda }\;d\lambda }{\int_{\lambda_{\min}}^{\lambda_{\max}}\;S_{\lambda }\;d\lambda },$$ (2)

where *λ* refers to the wavelength; *λ_min_* and *λ_max_* represent the lower and upper bounds, respectively, of the selected waveband; and *ρ_λ_*, *τ_λ_*, and *S_λ_* are the spectral reflectivity, spectral transmissivity, and radiation source flux at wavelength *λ*, respectively. *ρ_λ_*, *τ_λ_*, and *S_λ_* can be manually measured by using spectroscopic devices, or by consulting available spectral libraries such as the Ecological Spectral Information System [31]. *C_λ_* is the normalized spectral sensitivity of the camera sensor for a given wavelength (e.g., *C_λ_* = 1 means that the camera can detect 100% of incoming radiation at that wavelength). To reduce computational complexity, the integration is performed in a pre-processing step, and *ρ*, *τ*, *ρ_o_*, and *τ_o_* for each primitive are stored in GPU memory during ray-tracing.

A limitation of this ray-tracing model is that the incident spectral energy flux distribution is calculated based on that emitted by the source, *S_λ_*, which may be different from the actual spectral flux reaching a leaf if there is multiple scattering. This loss of accuracy for multiply scattered radiation is a compromise in favor of efficiency gained by the reverse ray-tracing approach.

#### Simulated camera

**Fig. 2.**
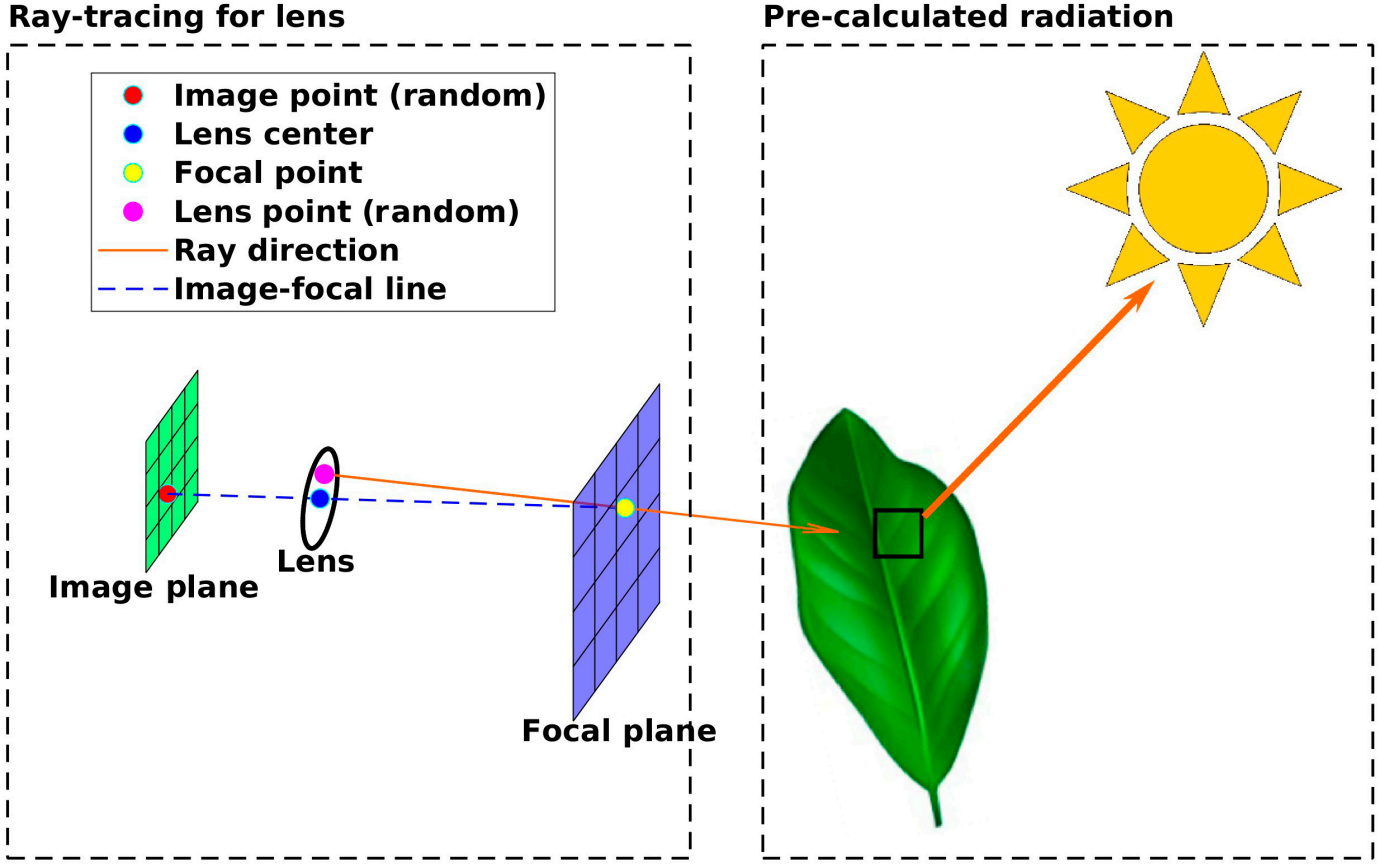
Schematic illustration of the ray-tracing-based method for camera simulation based on a thin lens model. Right panel: Radiation sources are sampled by each leaf surface element (solid black box) using a reverse ray-tracing approach to determine scattered radiation fluxes based on the model of Bailey [19]. Left panel: The camera model launches rays randomly sampled on the lens and passing through the focal plane, which queries scattered radiative fluxes and unique identifiers of surface element it hits.

A thin-lens camera model [32] is employed to sample radiation that is reflected, transmitted, or emitted from geometric elements based on radiation fields computed by the radiative transport model described in Discussion (Fig. 2). A thin-lens camera model can represent perspective and focus, but does not explicitly represent lens distortion. The model represents distortion through a calibration process outlined in Discussion. The user-specified parameters for the camera model are the horizontal field of view (HFOV), image resolution, lens diameter, focal plane distance, sensor size, the position of the camera, and the orientation of the camera. The goal of the model is to estimate the radiative flux sensed by each pixel in the simulated camera over a given wave band.

The camera model launches rays from simulated camera pixels in a pattern that reproduces image perspective and focus consistent with the camera input parameters listed above. In order to determine the origin and direction of these rays, the model considers 3 parallel planes: image plane, lens principal plane, and focal plane. The image plane is subdivided into pixels, with physical size equivalent to the sensor size, and distance from the lens principal plane being determined by both the sensor width and the HFOV. The focal plane aligns with the image plane, and its size is a function of the distance between the image and the lens, the distance between the lens and the focal plane (focal length), and the sensor size. The vector originating from the center of the image plane and passing through the center of the lens and focal plane defines the camera viewing direction. The model selects a predefined number of points on the lens based on jittered random sampling to compute the ray origin (pink point in Fig. 2). Simultaneously, it randomly samples an equal number of points on the pixel (red point) and calculates the corresponding points on the focal plane (yellow point). These focal plane points are determined by drawing lines (blue dotted line) through the sampled points on the pixel and the lens center. Then, the directions (red arrow in Fig. 2) of these rays can be determined from the points on the lens to the points on the focal plane. If an object is not located on the focal plane and the lens has a diameter greater than zero, the image will be blurred due to the point spread function. If the lens diameter is set to close to 0, the camera model will become a pinhole model. In this case, all objects, whether they are on the focal plane or not, will be in focus, and the simulated image will be sharp and free of blur.

#### Ray-tracing procedure to calculate camera pixel fluxes

Modeled pixel fluxes are calculated by combining the GPU-accelerated reverse ray-tracing method for modeling overall radiation transport [19] with the camera-based ray-tracing sampling described above. Rays are launched from randomly sampled locations on primitive element surfaces toward radiative sources in the case of external sources, and in a hemispherical pattern in the case of longwave sources. Details on how the primitives and sources are sampled to determine the ray origins and directions are given in [19]. A flux value is assigned to the ray based on the intensity of the source being sampled for external sources, or based on the emitted flux for longwave sources (and normalized by the number of rays per primitive) [19]. Some fraction of this ray energy is either absorbed by the primitive, reflected, or transmitted. The reflected and transmitted radiation is stored in “to-be-scattered” buffers for each side of the primitive, one set based on *ρ* and *τ*, and another set based on *ρ_o_* and *τ_o_* corresponding to radiation scattered to the camera. In the current implementation, it is assumed that reflection and transmission are Lambertian, and thus all scattered energy is aggregated together in the buffers regardless of its incident angular distribution. Accordingly, specular reflection is also currently neglected. Unlike the previous radiation model implementation in Helios, in the latest implementation (v1.3.0), calculations are performed for all radiation bands simultaneously without separate ray-tracing passes for each band, which reduces the model runtime considerably when there are many bands.

At this point, the camera ray-trace has not been performed and no energy has reached the camera—there is only energy residing in the “to-be-scattered” buffers for each primitive, which now needs to be sampled by the camera. The camera ray-trace is then performed to determine the amount of the “to-be-scattered” energy that reaches each camera pixel. If a camera ray intersects an object, its scattered energy (based on *ρ_o_* and *τ_o_*) is queried in order to determine the radiative flux sensed by the camera pixel. At the end of each scattering iteration, the “to-be-scattered” buffers for all primitives are set to 0. This energy scattering process is then repeated, starting with the general radiative transport ray-trace to calculate “to-be-scattered” fluxes, then the camera ray-trace to accumulate the additional scattered energy. This proceeds iteratively until the amount of scattered energy becomes arbitrarily small.

The result of the above calculation procedure is the sensed radiation flux for each pixel in the simulated camera for each wave band considered (in addition to absorbed radiative fluxes for each primitive element).

#### Radiation transfer model verification

The RAMI On-line Model Checker (ROMC, https://romc.jrc.ec.europa.eu/_www/) was used to verify the modeled radiation transport among canopies and the radiation received by the simulated camera sensor. ROMC [33] provides a means for assessing the accuracy of user 3D radiation transfer models by comparing them against reference models selected during the third phase of the RAdiation transfer Model Intercomparison (RAMI) exercise [34]. ROMC advises using results from its “validate” mode for the assessment of a model’s performance in scientific research. Accordingly, we selected the widely used *brfpp_uc_sgl*, *brfpp_co_sgl*, *brfop*, and *fabs* measurements, and all scenes under these measurements to validate the present ray-tracing model. A case with real-world canopy architecture derived from the Wellington Citrus Orchard from RAMI IV [35] was also selected for validating model performance. Further details regarding the ROMC and RAMI actual case verification settings can be found in Section S1.

### Image annotation

The current framework supports automatic image annotation (allowing the assignment of traits to individual pixels) that incorporates multiple traits at 2 distinct levels. The annotation process starts by determining the unique identifiers of geometric element(s) contained in every pixel of the simulated image. As is the case for real image annotation, only the closest object to the camera in each pixel is considered for labeling. Once the object identifiers for each pixel are known, any information in Helios about these primitives can be queried and used to generate “labeled” images. More specifically, this is achieved by using the element unique identifier contained in the pixel to look up “primitive data” values within Helios [25].

To identify the primitive label for a pixel in the image, only one ray is launched from the lens center and passes through the center of the corresponding pixel on the focal plane (Fig. 2). The label of the nearest primitive intersected by this ray is returned. Since the class label, such as leaf ID, cannot be mixed, a fuzzy state pixel is not considered for image annotation.

There are generally 2 classes of traits that may be specified in Helios for generation of annotated images. The first level of traits are user-specified such as plant height, leaf chemical concentrations, plant or leaf ID for object detection, etc. For these types of traits, users set particular primitive data values for all primitive elements in the scene (e.g., plant ID). This would then allow for generation of an image annotated by all of these data values.

The second level traits are based on data values computed within Helios. Examples of such types of values are net photosynthetic flux, leaf angle, etc., which are either computed based on a model plug-in in Helios or automatically assigned geometric properties.

Thermal and depth images are also generated using the same principle as labeling, while the labels are temperature and distance, respectively. Note that thermal images can also be generated based on the emitted radiation flux to be consistent with the quantity actually measured by real thermal cameras. More details can be found in Section S6.

### Camera calibration

#### Distortion recovery

Images captured by cameras are normally distorted due to lens aberration or sensor misalignment. In most remote and proximal sensing applications, the objective of image pre-processing is to remove these distortions [13,36,37]. In contrast, the ray-tracing model described in the “Methods” section generates perfect undistorted images as a result of the thin lens model. In order to recover the image distortion to make the output synthetic images resemble real images, simulated lens distortion is added to the synthetic images.

The radial and tangential distortion of images can be described mathematically as [38]:

$$\begin{array}{c} \begin{array}{c} \delta {\hat{u}}_{i}=u_{i}\left(p_{1}{r_{i}}^{2}+p_{2}{r_{i}}^{4}\right)+2p_{3}u_{i}v_{i}+p_{4}\left({r_{i}}^{2}+2{u_{i}}^{2}\right),\\ \delta {\hat{v}}_{i}=v_{i}\left(p_{1}{r_{i}}^{2}+p_{2}{r_{i}}^{4}\right)+2p_{4}u_{i}v_{i}+p_{3}\left({r_{i}}^{2}+2{v_{i}}^{2}\right), \end{array} \end{array}$$ (3)

where (*u_i_*, *v_i_*) represents the original position of the *i*th pixel in the image coordinate (the principal point is the center of the image), $\left(\hat{u},\hat{v}\right)$is the pixel position after distortion, *p*_1_ and *p*_2_ are the radial distortion coefficients, *p*_3_ and *p*_4_ are the tangential distortion coefficients, and ${r_{i}}^{2}={u_{i}}^{2}+{v_{i}}^{2}$. This distortion mechanism is integrated into the camera model by adjusting the location of the respective pixel in the original image generated by the ray-tracing model. Consequently, the locations of all pixels are rearranged to produce the distorted output image. The verification process of the distortion is described in Section S2. If all coefficients are set to 0, the distortion will not be applied, leaving the image in its original, undistorted state.

#### Camera spectral response calibration

The quality of simulated images is strongly dependent on the input camera spectral responses. However, the response spectra for many cameras are not easily accessible online, and if available from measurements, the spectral response of the camera sensor may differ from that of the actual image due to lens effects and internal software corrections applied by the camera. Additionally, there may be some inaccuracy in the specification of surface reflectance/transmittance and light source spectra based on spectrometer measurements. Therefore, it may be necessary to apply a calibration based on output images rather than simply using the spectral response of the sensor specified by the manufacturer. This correction was applied by determining the effective camera spectral response based on the calibration procedure outlined in Section S2.

To calibrate the camera, the target (physical) camera first captures an image of a reference material with a known spectral reflectance and transmissivity directly opposite to the camera in a blank and open space under a lighting condition (such as sunlight without cloud cover). This ensures that the captured image only includes the reference material and the ambient lighting, without any additional objects or obstructions that could affect the calibration process. Therefore, the obtained image value of the material *m_real_* should be equal to its *ρ_o_* across a given image channel. Ideally, a color calibration card with a wide range of color patches should be used, such as the DGK Color Card (DGK Color Tools, Boston, Massachusetts, USA) chosen for this study. Users can also customize the object color values for greater flexibility in color calibration. In other words, it allows for the simulation of the camera spectral response without an actual target camera. The details of the calibration process and verification method are given in Section S2.

### Model of leaf optical properties

*ρ_λ_* and *τ_λ_* in Eq. 1, used for calculating surface radiative properties, can be specified through manual measurements or predicted by leaf optical models. Leaf optical models PROSPECT-D [28] and PROSPECT-PRO [29] were integrated within Helios in order to link plant function to simulated sensing measurements. The original PROSPECT model developed by Jacquemoud and Baret [39] is a leaf tissue radiative transfer model that characterizes the optical properties of plant leaves across the solar electromagnetic spectrum. This method originates from the plate model put forth by Allen et al. [40]. It assumes that a leaf consists of a pile of *N* uniform layers divided by *N-1* air gaps. The integration with PROSPECT-D and PROSPECT-PRO enhances the current framework, enabling the generation of plant images that have corresponding distribution maps of leaf chlorophyll, carotenoids, anthocyanins, dry mass, water, protein, and carbon-based constituent concentrations.

### Example plant phenotyping applications

#### Bean leaf detection

**Fig. 3.**
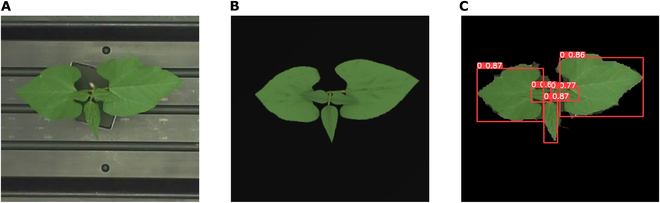
Example images from the bean leaf detection case. (A) Real bean RGB image from MSU-PID; (B) synthetic bean RGB image; (C) real bean image with background removed, labeled by a model trained on 105 synthetic images (annotations are labeled with the class type [0 represents a leaf] and a subsequent IoU value).

To investigate the potential benefits of including synthetic images in the machine learning model training pipeline, we considered an example phenotyping application aimed at detecting bean leaves within real RGB plant images. A publicly available dataset from the Michigan State University Plant Imagery Database [13] was used in this test. This dataset is composed of RGB images of 5 early-stage bean plants, with each plant having 35 leaf label maps at various growth stages. It should be noted that these segmentation maps were extracted from the fluorescence images given in the dataset rather than the RGB images. However, due to slight positional differences between the RGB and fluorescence sensors, these segmentation maps were translated and rescaled to align with the RGB images, which may introduce a minor amount of error. Specific details regarding the light source spectral distribution and the camera response spectra were not known, and reasonable values were determined through trial and error. The simulated scene was created with 9 Cree XLamp XHP70.2 LED spherical light sources. Initial camera parameters were assumed to correspond to a Basler ace acA2500-20gc RGB camera (Basler, Ahrensburg, Germany), which was then calibrated under the LED lights. Leaf radiative properties were specified by manually determining an appropriate range of concentration parameters in the leaf optical model. For the bean leaf plants in this dataset, which are small and in early stages of growth, the Helios “Plant Architecture” plug-in was utilized to construct the plant geometry. This model offers flexible parameters, allowing for the construction of plants ranging from the main stem to sub-stems, and setting the number and size of leaves per petiole. Initially, we created bean plant models at several growth stages that closely resembled the real images (Fig. 3). Subsequently, we randomly varied the rotation of leaves and plants along 3 axes, as well as the size of leaves, within empirically defined ranges to generate a collection of synthetic images representing different growth stages. The triangular meshes comprising the bean leaf prototypes were first constructed in Blender software (Blender Foundation; https://www.blender.org), which are then scaled, translated, and rotated appropriately within the plant architecture model (see also Section S3). The radiation simulations utilized settings of 5 scattering iterations and 3 diffuse rays per primitive.

For both real and synthetic images, the backgrounds were removed by converting the RGB images to the HSV color space and filtering based on threshold values. The deep learning model “YOLOv5s” [41], a member of the “You Only Look Once” (YOLO) model family that is popular for object detection tasks, was used for the detection.

By employing varying numbers of real and synthetic images for training, the efficacy of the synthetic imagery model was analyzed. Across different tests, the model size remained consistent, and the training parameters, such as learning rate, were also kept uniform. Model training was conducted for 150 epochs with an image size of 256 × 256 pixels. Various models trained based on 35 real images (plant 1), 35 real images plus 35 synthetic images, 35 real images plus 70 synthetic images, 35 real images plus 105 synthetic images, 105 synthetic images, and 140 synthetic images were tested on the other 140 real images (other 4 plants). The batch size equaled to the total number of training images. Model performance was evaluated using the mean average precision at an intersection over union (IoU) threshold of 0.5 (mAP50).

#### Unsupervised strawberry detection

A second case was considered in which the goal was to detect visible strawberries in images of strawberry plants in the field. The openly available dataset named “strawberry.00” was used, available at https://universe.roboflow.com/skripsie/strawberry.00. This dataset is composed of 450 annotated images, 59 and 391 of which were utilized for training and testing, respectively. The image dataset contains some unripe strawberries that are partially or fully green, and only fully red or “ripe” berries are annotated. This presents a challenge for the model because it cannot simply detect strawberries, or red regions in the images. This also creates some ambiguity for a human labeler as to what constitutes a fully red berry.

For this study, we generated synthetic images (as shown in Fig. S9) for unsupervised training of the YOLOv5s model. The camera model used to create the real image dataset was not known, so it was assumed that the camera was similar to a Nikon B500 camera, and calibration was then performed according to the procedure described in the “Camera calibration” section. 3D strawberry plant geometry was created using the “Canopy Generator” plug-in in Helios. Specifically, the plant height, strawberry radius, and leaf length were initially set within the ranges of 0.2 to 0.4 m, 0.04 to 0.08 m, and 0.05 to 0.1 m, respectively. Subsequently, the entire plant geometries were scaled down by a factor of 0.8, resulting in the actual sizes (units in meter) of these elements being 0.8 of their original settings in the simulated scene. This adjustment was necessary because the “Canopy Generator” offers quick configuration options but does not allow for adjustments to individual stem and petiole settings. To approximate real-world plant sizes and make the geometry appear closer to actual examples, scaling was employed. The number of stems per plant and the number of strawberries per stem were configured to 15 and 1 to 3, respectively.

The radiation simulations utilized settings of 5 scattering iterations, and the number of diffuse rays per primitive was 3. The strawberry fruit spectral reflectivity was assigned based on published data from Weng et al. [42]. Berry reflectivities were randomly scaled between 0.95 and 1.05 for the 610- to 700-nm waveband (red band). As there are some fully green or partially green berries in the original dataset, the surface reflectivity of green berry surfaces was specified using leaf optical model simulations (low input chlorophyll and carotenoid concentrations). The greening starts at the top of the berries, and the extent of this green portion was randomly set, while the remaining portion retained the strawberry red reflectivity. The leaf radiative properties were set using the same strategy as in the bean case. Strawberry plants were illuminated by simulated sun, and zenith and azimuth angles of the sunlight were randomly set during simulated image generation. Real images from the original testing set of the “strawberry.00” dataset were used to creating background-only images. The center parts of these images containing the plants were manually removed (as shown in Fig. S9a). Some background images containing unscreened residual strawberry patterns were excluded from selection, resulting in 50 background images chosen for training.

Model training was conducted for 60 epochs with an image size of 704 × 544 pixels. Models were trained and tested using different combinations of synthetic and background images: 50 synthetic images, 100 synthetic images with and without an additional 50 background images, 200 synthetic images with and without an additional 50 background images, and 300 synthetic images with and without an additional 50 background images. These models were then evaluated on the 391 real images. The batch size was adjusted based on the number of training images to ensure that the number of training iterations in each epoch is 3. Model performance was also evaluated using the mAP50.

## Results

### Radiation transfer model verification

**Fig. 4.**
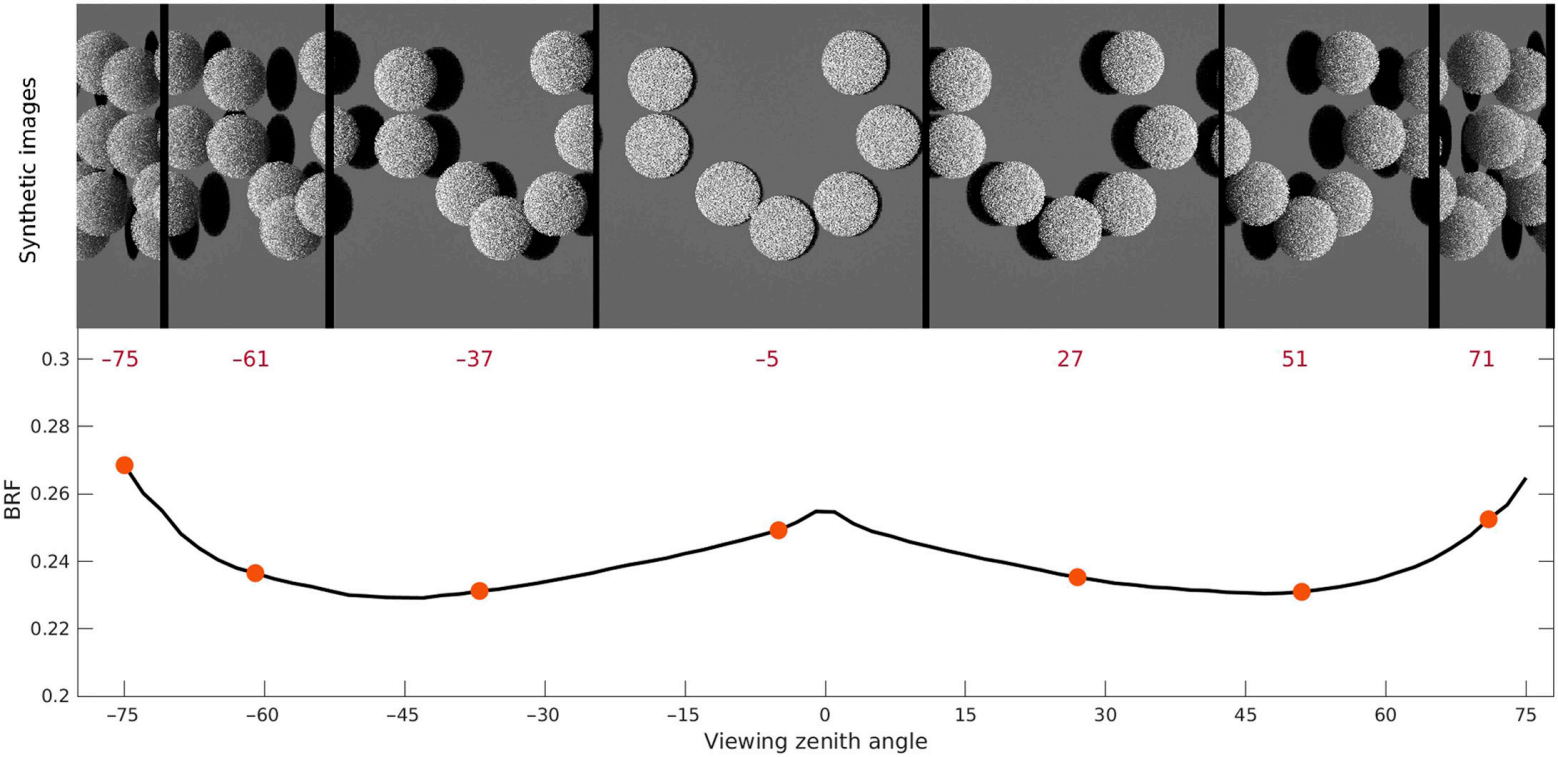
The bi-directional reflectance factor (BRF) curve of experiment HET51_DIS_UNI_NIR_00 measured in the ROMC case *brfop* and corresponding synthetic images captured by simulated cameras at multiple viewing zenith angles (red numbers below images).

Table 1 displays the SKILL scores for the RAMI verification tests *brfpp_uc_sgl*, *brfpp_co_sgl*, *brfop*, and *fabs*, which are 98.00, 92.65, 97.52, and 99.98, respectively. Figure 4 presents the simulated bi-directional reflectance factor (BRF) (black curve) for the measurement of *brfop* of experiment HET51_DIS_UNI_NIR_00 and the output images captured by the simulated radiation cameras at varying viewing zenith angles (red numbers) used to compute the BRF. The SKILL score of *fabs* indicates that the ray-tracing model is excellent at describing the radiation absorbed by objects in the scene, and SKILL scores of *brfpp uc sgl, brfpp co sgl,* and *brfop* indicate that the simulated camera can correctly capture the reflected radiation fluxes (more detailed results of *brfop* can be found in Section S1). The scattering iteration number used for verification was set to 20 for *brfop*. It was observed that the BRF tends to converge around 15 to 20 iterations, as shown in Fig. S2. The need for a relatively high number of iterations is due to the high complexity of the test scene and large differences in surface reflectivity. The number of diffuse rays per element has a minimal impact on the BRF simulation, with nearly identical results obtained for 1, 100, and 1,000 rays per primitive. This is attributed to the reverse ray-tracing approach ensuring every element is sampled, and because the total number of primitives in the ROMC test scenes is high (approximately 3.08 million for experiment HET01_DIS_UNI_NIR_00). Consequently, the total number of rays in the scene used for running the ray-tracing model is always sufficiently large. The outcome of the actual scene case from RAMI IV, as obtained by the current ray-tracing model, falls within the range of the RAMI IV benchmark models (as depicted in Fig. S3). This demonstrates that the present ray-tracing model is effective in handling complex actual scenes.

**Table 1.** Results of sub-model verification

	Results	
Ray-tracing model	SKILL (*brfpp_uc_sgl*)	98.00
	SKILL (*brfpp_co_sgl*)	92.65
	SKILL (*brfop*)	97.52
	SKILL (*fabs*)	99.98
Camera calibration	*R*^2^ (distortion recovered)	0.930
	*R*^2^ (undistorted)	0.919
	*R*^2^ (color calibrated)	0.903
	*R*^2^ (color uncalibrated)	0.864

The *R*^2^ value for checker square corner positions between simulated checkerboard images without distortion and MATLAB built-in reference images was 0.919, improving to 0.930 when distortion is applied (Table 1). Figure S4 demonstrates the current framework’s proficiency in accurately recovering distortion. Consequently, the ability to recover plant image distortion within the current framework is likely to improve its ability to reproduce real proximal images and aid machine learning model training.

**Fig. 5.**
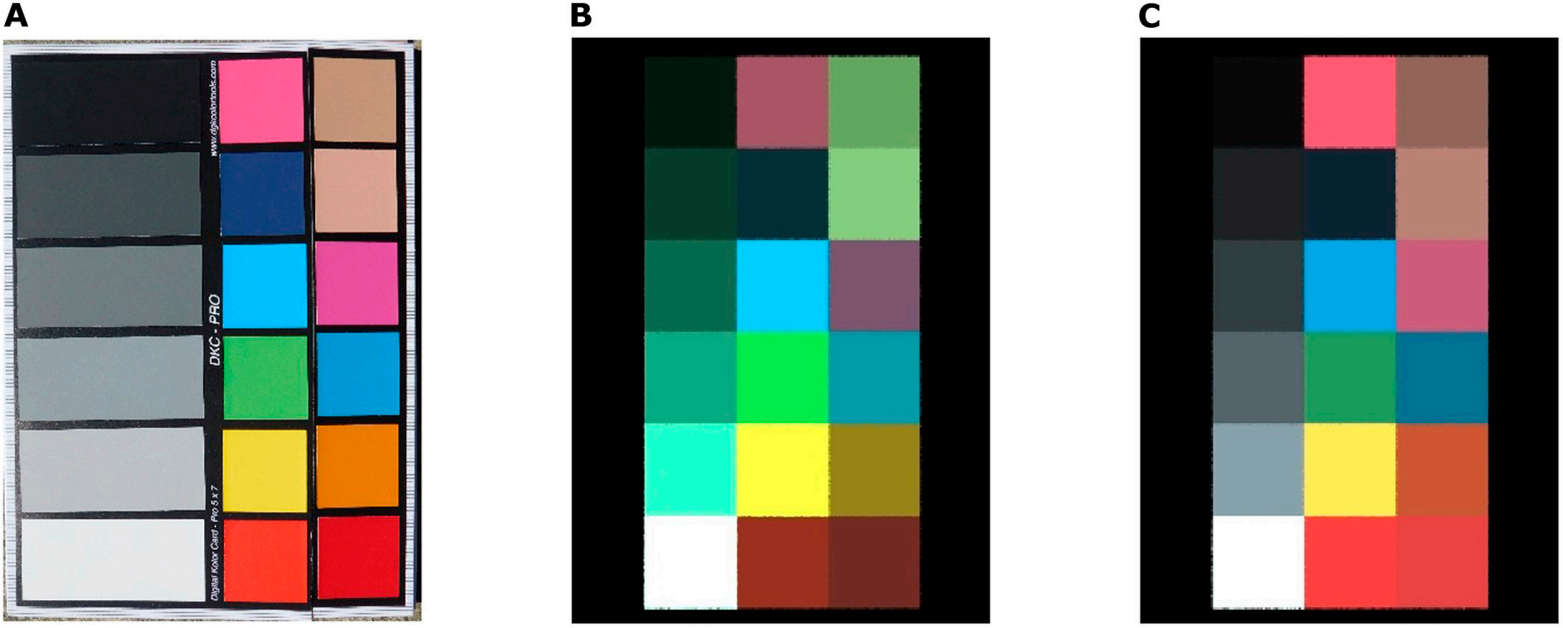
Calibration of camera spectral response. (A) Real image of DGK Color Card captured by a Nikon B500 camera, (B) uncalibrated synthetic color board image captured by simulated Nikon D700, and (C) calibrated synthetic color board image captured by simulated Nikon B500 under sunlight.

Figure 5 shows the color board image (captured under solar conditions in the real world by a Nikon B500 camera) used as a reference for color values, as well as the 2 synthetic color board images created using calibrated Nikon B500 and uncalibrated Nikon D700 cameras, respectively. The corresponding calibrated and uncalibrated camera response spectra can be found in Fig. S5. It is visually evident that the color board in the image captured by the calibrated camera more closely resembles the real image. The *R*^2^ value for the calibrated color values is 0.903, while the uncalibrated color values have an *R*^2^ of 0.864. It is noteworthy to highlight that when users perform calibration without a physical target camera (for instance, by assigning reference color values derived from online images), they create a completely new simulated camera spectral response, which is designed to facilitate the rapid utilization of the model. An example of synthetic plant images taken by a calibrated Nikon B500 camera and uncalibrated Nikon D700 can be found in Fig. S6.

The results from sub-model verification demonstrated consistency in the implementations for simulating the radiation field. Example synthetic plant images are shown in the following section.

### Synthetic image examples

**Fig. 6.**
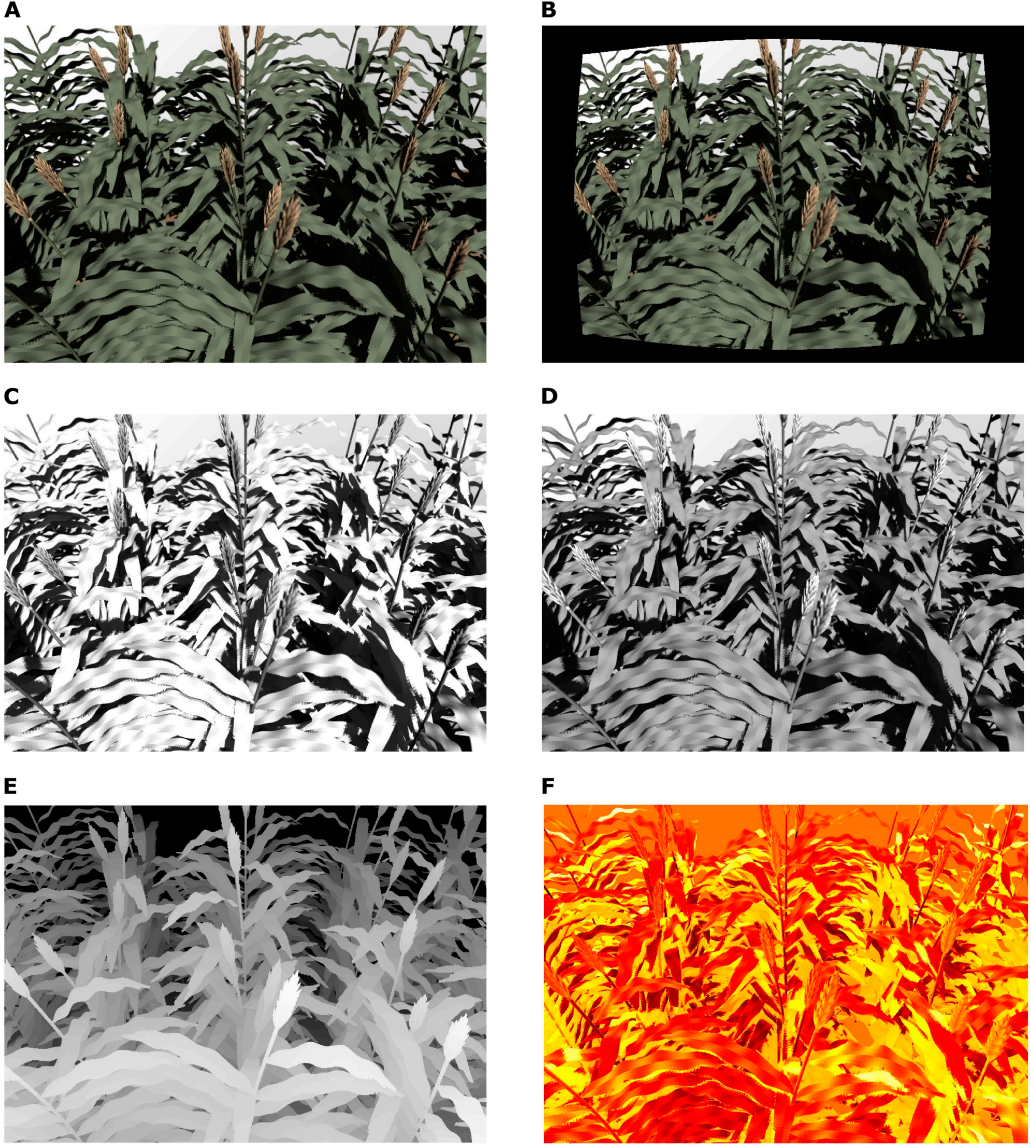
Synthetic images of sorghum plants illuminated by the sun. (A) Raw output RGB image, (B) RGB image after distortion recovery, (C) 980-nm NIR image, (D) 550-nm VIS image, (E) depth image, and (F) thermal image. Note that the black gaps around the image border in (B) result from application of the image distortion recovery procedure. In (E), the color scale ranges from white (closest to the camera) to black (furthest from the camera). In (F), the color scale ranges from black (coldest) to yellow (hottest). The camera focal plane distance, HFOV, and diameter of the lens are 3 m, 54°, and 0.02 m, respectively. The 550-nm VIS image and 980-nm NIR image are captured by a simulated multispectral camera (Spectral Devices Inc., London, Ontario, Canada).

**Fig. 7.**
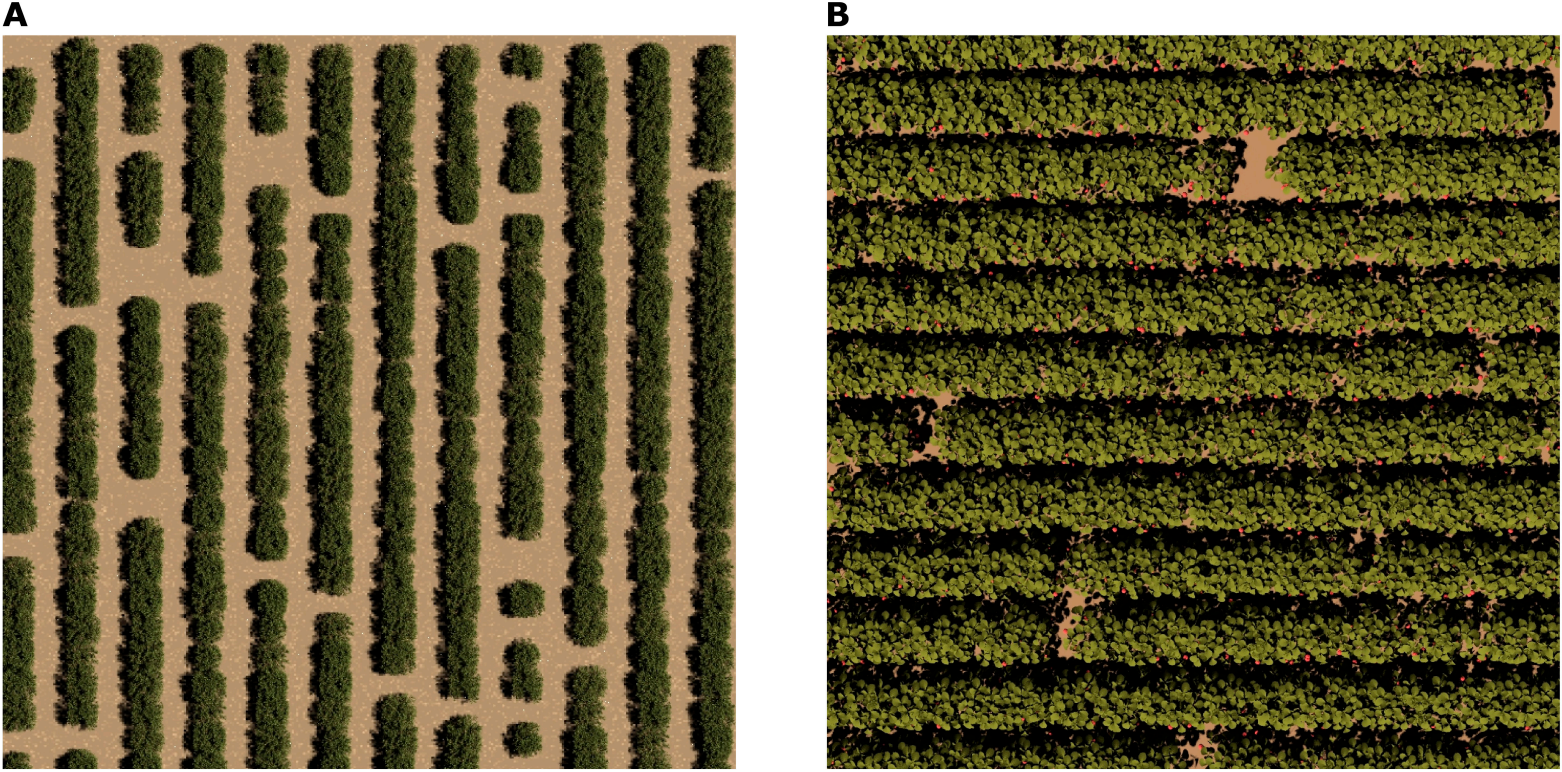
Example large-scale simulated images. (A) Synthetic RGB image of part of the “Wellington Citrus Orchard” (extent 50 × 50 m^2^) illuminated by a solar light source. The camera focal plane distance, HFOV, and diameter of the lens are 100,000 m, 0.143°, and 0.01 m, respectively. (B) Synthetic RGB images of strawberry plants illuminated by a solar light source (extent 8 × 8 m^2^). The camera focal plane distance, HFOV, and diameter of the lens are 11.5 m, 34.2°, and 0.01 m, respectively.

Figure 6 displays synthetic raw RGB (Fig. 6A), distorted RGB (Fig. 6B), 980 nm (Fig. 6C), 550 nm (Fig. 6D), depth (Fig. 6E), and thermal (Fig. 6F) images of sorghum plants under direct sunlight. Qualitatively, the model is able to generate high-quality images that are visually similar to real images. The near-infrared (NIR) (980 nm) image reveals different features from the RGB images, as is expected given the differences in surface radiative properties across these bands. Figure S8 provides an example of the same sorghum plant scene under a solar source with various zenith and azimuth angles, illustrating distinct differences in lighting patterns. This feature can be used for time-dependent plant physiological studies. In addition to small-scale images, Fig. 7 displays large-scale images of strawberry plants and part of the “Wellington Citrus Orchard” from RAMI IV [35]. This illustrates the framework’s capability to produce synthetic images on a large scale, contributing to drone-based plant phenotyping and physiological analysis.

**Fig. 8.**
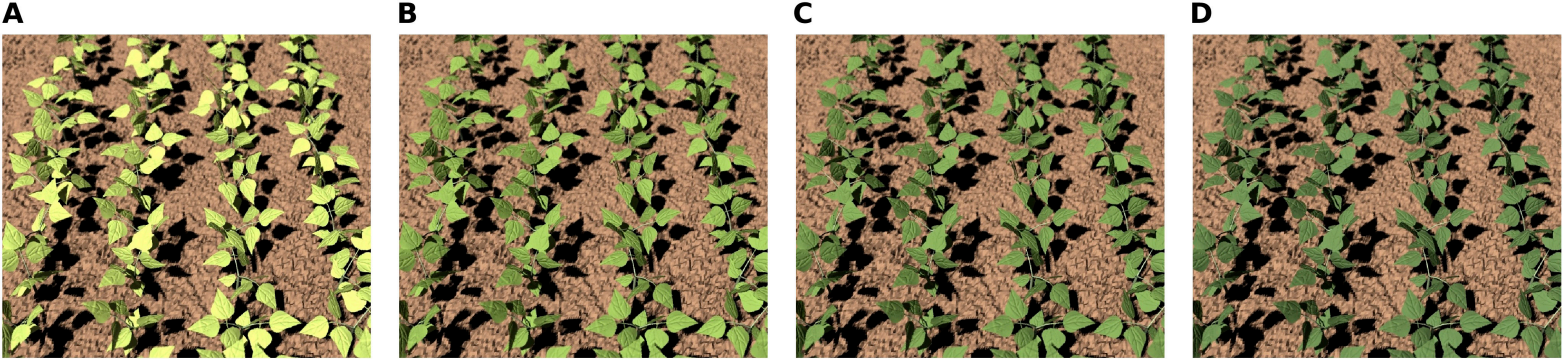
Synthetic bean RGB images with varying input leaf concentration of chlorophyll (from A to D: 10, 20, 30, and 40 μg/cm^2^) and carotenoids (from A to D: 2.5, 5, 7.5, and 10 μg/cm^2^). Other leaf properties remained constant: the number of elementary layers *N* = 1.5, anthocyanin concentration was 1 μg/cm^2^, equivalent water thickness was 0.015 g/cm^2^, and dry mass per area was 0.009 g/cm^2^. The camera focal plane distance, HFOV, and diameter of the lens are 1.55 m, 9°, and 0.02 m, respectively.

Figure 8 shows simulated RGB images of a bean crop with varying optical properties output from the PROSPECT model with varying input leaf chemical concentrations. Specifically, Fig. 8 illustrates changes in chlorophyll concentration between 10 and 40 μg/cm^2^ and carotenoid concentration between 2.5 and 10 μg/cm^2^ (the ratio of chlorophyll to carotenoid concentrations was set to 4). Overall, these images illustrate the clear impact of varying leaf chemical properties on the synthetic images.

**Fig. 9.**
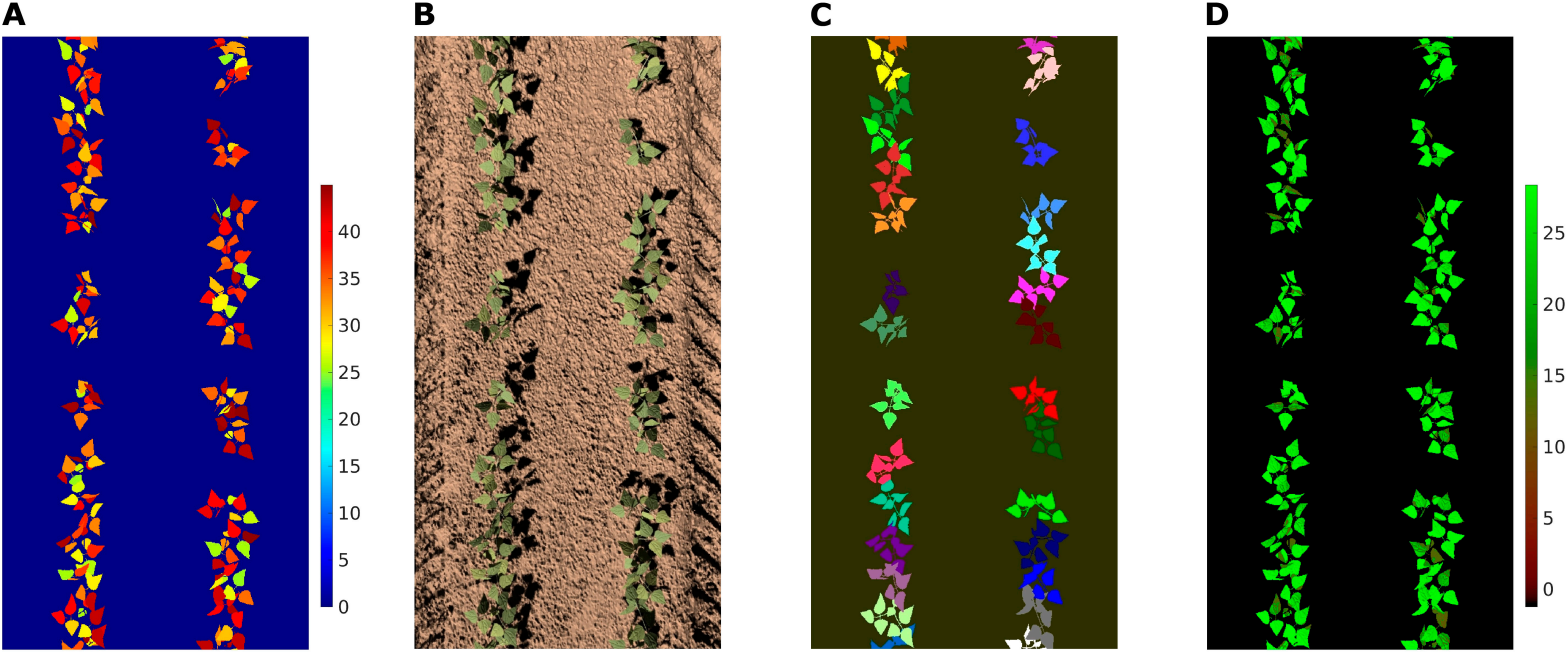
Example annotated synthetic images of bean plants. (A) Distribution map of leaf chlorophyll concentration (color scale given in units of μg/cm^2^), (B) synthetic RGB image, (C) plant segmentation map, and (D) distribution map of net photosynthesis (color scale given in units of μmol m^−2^ s^−1^). The camera focal plane distance, HFOV, and diameter of the lens are 1.35 m, 11.5°, and 0.02 m, respectively.

Figure 9 provides an example of automatic image annotation for bean plants. Figure 9A illustrates the randomly specified chlorophyll concentrations ranging from 25 to 45 (the ratio of chlorophyll to carotenoid concentrations was set to 4). The leaf chlorophyll values were input to the PROSPECT model in order to calculate leaf reflectivity and transmissivity. The ray-tracing model was then run based on these properties and finally output bean plant RGB image (Fig. 9B). Figure 9C illustrates the associated plant segmentation map, with each color representing a unique plant, which could, for example be used to generate bounding boxes for object detection or masks for semantic segmentation. Figure 9D presents the distribution map of the net photosynthetic rate, calculated using the Farquhar, von Caemmerer, and Berry (FvCB) model [43]. For this example, the *V*_*cmax*25_ (maximum carboxylation rate), *J*_*max*25_ (maximum electron transport rate), and *R*_*d*25_ (dark respiration rate) at the reference temperature of 25°C required by the FvCB model were empirically calculated (Section S4). These images demonstrate the framework’s ability to link various verified components and generate annotated images, which could be utilized for a range of plant phenotyping applications. However, for users requiring more realistic images and corresponding distribution maps that closely mimic real-world plants, field measurements of model-required parameters such as chlorophyll concentrations, *J*_*max*25_, *V*_*cmax*25_, *R*_*d*25_, and environmental factors are necessary.

Details of the setup for synthetic images described in this section can be found in Section S3.

### Example plant phenotyping applications

#### Bean leaf detection

Training the bean leaf detection model using only a small number of real images (35 images of plant 1) resulted in the poorest performance out of the cases considered (Table 2). Adding 35 synthetic images helped to markedly improve detection performance, while the further addition of more synthetic images resulted in diminishing returns. Table 2 shows that adding 70 synthetic images or 105 synthetic images had similar benefit. These results are better than those of models trained with 35 synthetic images, and they are higher than those trained with only 105 or 140 synthetic images, which had mAP50 values of 0.764 and 0.753, respectively. It was thus possible to obtain reasonable model performance when the model was never shown real annotated images during training (i.e., “unsupervised”). Figure 3C displays an example where all leaves were accurately labeled by the model trained exclusively on 105 synthetic images. Nonetheless, these results also illustrate that the benefits of adding synthetic images are not limitless, as variability is constrained by factors such as plant geometry and lighting conditions in the image generation settings.

**Table 2.** Results of bean leaf detection tests. Each test used a different number of real and synthetic images for model training. Agreement between the human-annotated and predicted leaf bounding boxes was quantified using the best mean average precision at an intersection over union (IoU) threshold of 0.5 (mAP50) and mAP50 at the 150th epoch.

Real images	Synthetic images	Best mAP50 (mAP50 at the 150th epoch)
35	0	0.685 (0.683)
35	35	0.760 (0.748)
35	70	0.772 (0.768)
35	105	0.775 (0.758)
0	105	0.764 (0.756)
0	140	0.753 (0.751)

#### Unsupervised strawberry detection

**Fig. 10.**
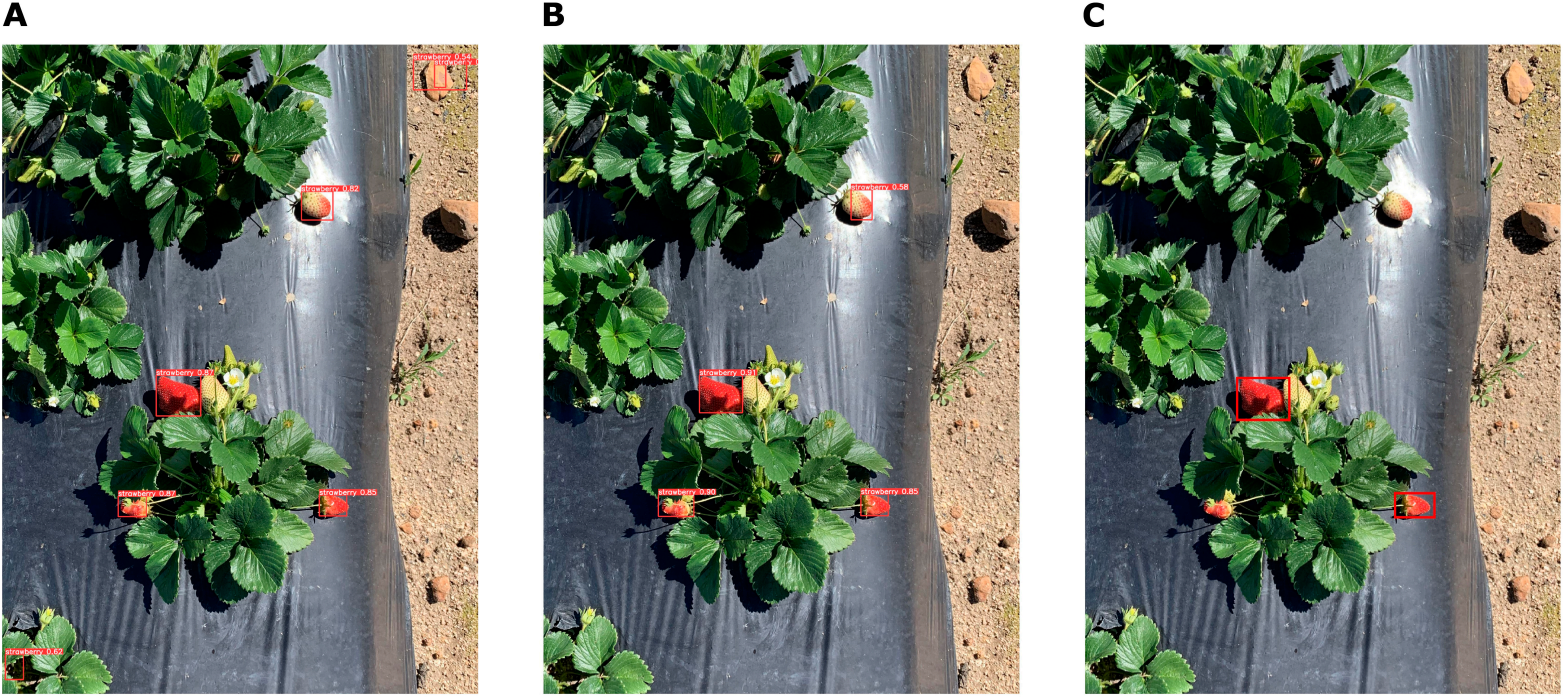
Example comparison of strawberry detection results based on one test image: (A) using the model trained with 100 synthetic images only, (B) using the model trained with 300 synthetic images and 50 background-only images, and (C) original label boxes from the dataset. Note that in the original dataset, only nearly full-red berries were labeled.

Table 3 presents the results of the strawberry detection tests using models trained with varying combinations of synthetic and real background images. Two types of mAP50 evaluations were conducted: one using the best model selected during training, and another using the model obtained after completing all 60 epochs of training. It is evident that increasing the number of synthetic images enhanced detection accuracy. Similarly to the bean results, adding more than 100 images does not enhance model performance in the absence of background images. Incorporating real background images can further improve the model performance (Table 3), a result that is clearly illustrated in Fig. 10. When the model was trained with 300 synthetic images and 50 background images, all strawberries in the example image shown in Fig. 10B were correctly detected.

**Table 3.** Results of unsupervised strawberry detection tests. Each test used a different number of real background and synthetic images for model training. Agreement between the human-annotated and predicted strawberry bounding boxes was quantified using the best mean average precision at an IoU threshold of 0.5 (mAP50) and mAP50 at the 60th epoch.

Synthetic images	Background images	Best mAP50 (mAP50 at the 60th epoch)
50	0	0.598 (0.582)
100	0	0.755 (0.748)
100	50	0.778 (0.772)
200	0	0.749 (0.715)
200	50	0.785 (0.771)
300	0	0.751 (0.728)
300	50	0.793 (0.780)

One source of error in the model was the presence of partially green berries. The criteria for berry labeling in the original dataset was unclear, as there were instances in which partially green berries were labeled. This caused some error in the model because it was trained to only detect fully red berries (as shown in Fig. 10A and B). In addition, some berries under the shadow of leaves were not labeled in the original dataset, yet most of them were detected by our model (such as the bottom right berry in Fig. 10). Therefore, the model’s performance may be higher than suggested by the obtained evaluation metrics. When no real background images were used in training, some small stones in the background were mistakenly identified as berries (Fig. 10A). This error was expected, given that the simulated images did not have any stones in the background, and the stones tended to have a red hue. However, adding some real background images (which do not require annotation) was able to mostly resolve this issue. Furthermore, some leaves were incorrectly classified as berries when the number of training images was small. Overall, the use of a substantial number of synthetic images combined with pure background images can achieve good results in this unsupervised strawberry detection case.

#### Speed of synthetic image generation

The total number of primitives in each bean plant scene ranged from about 113,000 to 127,000, depending on the number of leaves. The simulation speed for the bean plant was 11 s per scene with one image when using a laptop with an NVIDIA RTX A2000 (8 GB GDDR6) graphics card and a 12th Gen Intel Core i9-12950HX CPU. For the strawberry case, the time to generate one image was 13 to 14 s when using the same laptop. The total number of primitives in each strawberry scene ranged from about 200,000 to 220,000; this variation is caused by the number of leaves and berries, which change due to random variation.

Using a server-grade GPU considerably reduced the synthetic image generation time. In the strawberry case, the generation time was 3 to 4 s per image when using an NVIDIA A100 (80 GB) GPU coupled with an AMD EPYC 7713 32-Core Processor. Using an NVIDIA RTX A5500 (24 GB) GPU and AMD EPYC 7272 processor increased the time to 4 to 5 s per image. When processing a larger scene with over 2 million primitives, Table 4 shows the generation speed per image and host random-access memory (RAM) requirement when A100 and A5500 GPUs were used.

**Table 4.** Image generation speed and host memory usage for different large scenes using A100 and A5500 GPUs. The base scene used is the RAMI IV actual case (extent: 108.25 × 103.9 m^2^) with different number of trees added.

Number of primitives	Host RAM usage (gigabyte)	Generation speed (s/image)	GPU
2.15 million	7.55	48.6	A5500
2.15 million	7.54	36.2	A100
2.60 million	9.05	59.0	A5500
2.60 million	9.04	43.9	A100
3.75 million	12.95	86.5	A5500
3.75 million	12.91	61.4	A100

## Discussion

The present framework can effectively simulate a wide range of camera-based sensors in order to produce a large number of high-quality synthetic images of plant scenes that incorporate a range of labels, including canopy structure and leaf chemical properties under the specified light environment. The example application of bean leaf segmentation has initially demonstrated that synthetic images can directly serve as inputs to machine learning models designed for high-throughput plant phenotyping images to improve model training. It was also shown that the model could be trained exclusively based on synthetic images and perform well when applied to real images (Fig. 10). This illustrates a primary advantage of a realistic model that is close to reality, which is that there is a minimal impact on model training relative to real images. Integration of simulated data into model training may be considered a form of unsupervised learning, as it required no manual human annotation. The example application in strawberry detection further demonstrates the potential of the current framework in unsupervised plant phenotyping.

The background often contains noisy information, making it challenging to model in a simulated environment. Collecting background data is also considerably simpler and more straightforward than annotating plant traits. Results of the strawberry detection test suggested that creating a set of pure background images may be an effective strategy to enhance the performance of machine learning models trained with synthetic images (“Unsupervised strawberry detection” section).

Although the synthetic image sets used for illustration were relatively small, the GPU-accelerated model efficiency, combined with the “embarrassingly parallel” nature of synthetic image generation, enables the generation of massive simulated image sets. Each image is independent of another, which means that the images can be generated in parallel across a cluster of compute nodes. Given the runtime of around 4 s per image on a GPU server for the strawberry case, if we had a cluster of 25 nodes with comparable hardware, an image set of 1 million annotated images could be generated in less than 2 days. For larger scenes, adding more simulated cameras to capture images of the same scene from different angles can further enhance the efficiency of generating synthetic images. This would likely result in more efficient image generation because multiple images could be generated from a single scene, and thus overall time of scene generation and data transfer to the GPU is reduced. Additionally, as mentioned in the “Radiation transfer model verification” section, the number of diffuse rays can be reduced to 1 to achieve faster rendering speeds for large scenes.

The strawberry case also illustrated an interesting aspect of human annotation: the potential of using synthetic data as an assistant to human annotation. The process of human annotation is subjective and contains non-systematic bias, or may be limited by an individual’s skill. This is exemplified by annotation of the strawberry images, in which the definition of a fully ripe strawberry is not well-defined, and the annotators missed some strawberries within shadows (Fig. 10). Annotation of synthetic images is “exact” and based on strictly defined criteria. These results are promising in terms of illustrating the model’s capability to reduce or eliminate the amount of manually annotated data needed for model training.

Although the example applications focused on RGB imagery datasets, another important strength of the proposed modeling framework is that it can simulate other sensor modalities such as visible (VIS) multispectral and thermal imagery. The mechanism for generating VIS multispectral imagery, which includes modeling radiative properties and calibrating sensor spectral response, is exactly the same as for RGB imagery, given that RGB imagery can be considered a 3-channel multispectral imagery in the visible region. The generation of synthetic thermal images is conducted by integrating with other Helios components, which have been individually validated such as temperature/energy balance models [44]. A recent study [45] has also verified the point temperature values simulated using the present model. A comprehensive, pixel-by-pixel quantitative assessment of errors for an entire synthetic thermal image and net photosynthesis distribution map has not yet been conducted. Therefore, future work utilizing the model for thermal imagery-based phenotyping and analysis of net photosynthesis distribution should also include additional model validation.

Previous work has demonstrated the utility of incorporating synthetic imagery into the machine learning training process when the scope of available images is limited [26,46]. However, these studies were limited by the use of traditional computer graphics-based renderers that are not coupled to the underlying radiation physics or biophysics. This limits their application to object detection and segmentation tasks with RGB images. The image annotation capabilities of the present framework is not limited to only the traits shown in this paper, but also other commonly utilized plant traits such as plant height, canopy cover, stomatal conductance, and LAI [1,11,47]. Furthermore, the flexibility in utilizing simulated camera sensors can be beneficial for the fusion of multimodal data from different types of sensors, as all the sensor parameters are user-specified. Adding distortion recovery is also important, as distortion can influence plant phenotyping [48–50]. Overall, the framework can greatly enhance the efficiency and precision of high-throughput plant phenotyping, which is essential for agricultural and ecological research.

Apart from image synthesis, the current framework can act as a basis for investigating a variety of radiation-dependent processes, including photosynthesis, transpiration, and microclimate. Rapid measurement of traits associated with these processes has the potential to enhance our understanding of their distribution and interactions with canopy structure. In comparison to many analytical tools for plant phenotyping such as PlantCV [51] and HSI-PP [52] for plant image analysis, and AgML [22] and CropSight for data management [53], the current framework addresses the need for synthetic imagery and offers a solution for multimodal analysis tools. Compared to the LESS [20] and DART-Lux [27] models, the current framework offers integrated models for generating and modifying model geometry via Helios, making it especially suited for proximal remote sensing applications. Additionally, it provides pixel-by-pixel annotation and ability to couple with other biophysical models, which are not currently available in LESS and DART-Lux.

The framework implementation has limitations that suggest directions for future development. For instance, surface reflection and transmission are Lambertian in the model, and thus specular reflection and anisotropic scattering by surfaces are currently neglected. The lateral transmission of radiation within the leaf is not considered, implying that each primitive operates independently of others. Additionally, the non-leaf surface optical properties (e.g., stems, fruit, and soil) are set empirically, and thus lack interaction with physiological traits and chemical composition. Future enhancements will address these limitations, aiming to offer a more flexible and robust tool for plant image synthesis and radiative-based physiological modeling. While model efficiency allows for direct scaling to domains relevant to satellite images, the present modeling framework does not include atmospheric absorption and scattering such as in DART-Lux [27].

The modeling framework developed in this study is able to simulate radiation transport among objects, and ultimately, radiation is detected by a variety of simulated camera sensors. Realistic synthetic RGB and multispectral plant images were presented, demonstrating the framework’s ability to create images with distinct light and shadow features under various radiation sources. Additionally, the framework can generate large-scale synthetic images, facilitating the simulation of drone imagery. It can also produce images incorporating variation in leaf chemical properties, and generate precise annotations based on any user-specified or simulated data. The fully labeled synthetic spectral images can supplement machine learning model training by expanding reference datasets for predicting plant traits from real spectral images. Consequently, the framework is valuable for high-throughput plant phenotyping applications and has the potential to minimize the need for manually collected and annotated data in deep learning training. Finally, complex plant systems can be presented in a simple and intuitive manner, which is highly advantageous for radiative-based physiological modeling.

## Data Availability

The strawberry data that support the findings of this study are openly available in strawberry.00 at https://universe.roboflow.com/skripsie/strawberry.00. The Bean data that support the findings of this study are openly available in MSU-PID at https://www.cse.msu.edu/computervision/MVA15-MSU-PID.zip. Other data that support the findings of this study are openly available in Helios at https://github.com/PlantSimulationLab/Helios or available upon reasonable request.

## Supplementary Materials

Supplementary 1 Sections S1 to S6 Figs. S1 to S9 Tables S1 and S2 References [54–60]
