# Supplementary material for: Simulation of Automatically Annotated Visible and Multi-/Hyperspectral Images Using the Helios 3D Plant and Radiative Transfer Modeling Framework
Source: Plant Phenomics. 2024 May 30;6:0189. doi: 10.34133/plantphenomics.0189 (PMC11136674; doi:10.34133/plantphenomics.0189)
Supplement: Supplementary 1 — Sections S1 to S6 Figs. S1 to S9 Tables S1 and S2 References [54–60] [file plantphenomics.0189.f1.zip › SM.docx]

# Simulation of automatically annotated visible and multi/ hyperspectral images using the Helios 3D plant and radiative transfer modeling framework

# Supplementary 1. ROMC settings and verification results

Figure S1. ROMC summary result of *brfop* measurement

Two measurements were used for verifying the radiation with single scattering received by the simulated camera (Table S1): *brfpp_uc_sgl* and *brfpp_co_sgl*, which measure the Bi-directional Reflectance Factor (BRF) of soil and canopy in the principal plane, respectively. BRF is defined as the radiant flux exiting from a target area in a particular direction, passing through a planar reference surface, and normalized by the equivalent quantity leaving from an infinitely large Lambertian background via the same reference surface and for the same directions of illumination and reflection. A Lambertian background represents a perfectly reflecting isotropic surface according to ROMC defination. The reference surface, situated perpendicularly to the normal of the underlying surface, has defined lateral dimensions and elevation above the target area. The principle plane implies that when the sun azimuth angle is , the viewing azimuth angle corresponds to . The image size for the simulated cameras changed with zenith angle to ensure that only radiation fluxes that passed through the reference plane were collected. The distance between the canopies and the simulated camera was set to a very large value (100,000 m) to guarantee that the reflected radiation fluxes were nearly parallel and very close to the prescribed viewing zenith angle.

Another two measurements *fabs* and *brfop* (Table S1) were employed to measure the percentage of radiation entering the canopy via the reference plane that has been absorbed by the leaves excluding the soil, and BRF with multiple scattering in the cross plane, respectively. The cross plane is oriented perpendicularly to the principal plane, representing a viewing azimuth angle when the sun azimuth angle is . All experiment IDs are listed in Table S1. The ROMC website provides comprehensive information on scene details, including geometries, scene extent, radiative properties, and sun and viewing directions, which can be easily accessed by searching for these experiment IDs.

The SKILL score is the indicator of the model performance for all of these four measurements, which can be computed as:

(S1)

where denotes the Pearson correlation coefficient, and are the mean values of results obtained by present framework and the ROMC reference results, respectively, and and indicate the standard deviation of results. Various experiments containing both heterogeneous and homogeneous canopies under different sun zenith angles were randomly selected for the verification test.

Figure S1 presents the BRF simulated results (red curves) against the ROMC reference results of the *brop* case. Table S2 provides the ROMC experiment IDs employed for verifying the present ray-tracing model. For *fabs* and *brfop*, the radiation flux and scattering depth (iteration) were assigned values of 10 W/m and 20, respectively. For each element, the number of direct rays was set to 100, while the number of diffuse rays was set to 1,000, both of which are default settings in Helios. For the measurements *brfpp_uc_sgl*, *brfpp_co_sgl*, and *brfop*, 2-degree intervals of the view zenith angle were set, covering a range from -75 degrees to 75 degrees in the target plane. The camera resolution was set to , and a total of 100 rays were launched from each pixel on the camera image plane. The test scenes for these three measurements were duplicated to the left, right, up, and down of the target test scene used for observing. This was done to ensure radiative energy conservation in the observation plane. As a result, there are five replicated test scenes for each of these three measurements. Figure S2 shows BRF curves of experiment HET01_DIS_UNI_NIR_00 measured in the ROMC case *brfop* by using 5, 10, and 20 scattering iterations.

For the actual scene “Wellington Citrus Orchard" with experiment ID of HET14_WCO_090_B01_20 from RAMIV IV [36], Fig. S3 shows the BRF (*brfpp*) curves obtained by our ray-tracing model and the bench mark models provided by the RAMI IV website. The *brfpp* is similar to the *brfop*, while it sets viewing azimuth angle when the sun azimuth angle is . Detailed information is available on the RAMI IV website by searching the experiment ID: HET14_WCO_090_B01_20.

Table S1. Selected ROMC measurement IDs and experiments for verification

| Measurement | *brfpp_uc_sgl* and *brfpp_co_sgl* | *brfop* and *fabs* |
| --- | --- | --- |
| Experiment1 | HET21_DIS_UNI_RED_00 | HOM15_DIS_ERE_RED_60 |
| Experiment2 | HET11_DIS_UNI_RED_30 | HET01_DIS_UNI_RED_30 |
| Experiment3 | HET21_DIS_UNI_NIR_30 | HET51_DIS_UNI_NIR_00 |
| Experiment4 | HET01_DIS_UNI_NIR_00 | HET01_DIS_UNI_NIR_00 |

Figure S2. The Bi-Directional Reflectance Factor (BRF) curve of experiment HET01_DIS_UNI_NIR_00 measured in the ROMC case *brfop* by using 5, 10, and 20 scattering iterations.

Figure S3. The BRF (*brfpp*) curves of the actual scene “Wellington Citrus Orchard" obtained by RAMI IV bench mark models and the present ray-tracing model (Helios-RT). The details and reference of these benchmark models can be found in [36].

Table S2. ROMC scene settings for verification

611

| l X X X X X Experiment1 | Leaf reflectance | Leaf transmittance | Soil reflectance | Solar Zenith Angle [deg] | Extent [m] |
| --- | --- | --- | --- | --- | --- |
| HET21_DIS_UNI_RED_00 | 0.02 | 0.01 | 0.15 | 0 |  |
| HET11_DIS_UNI_RED_30 | 0.02 | 0.01 | 0.15 | 30 |  |
| HET21_DIS_UNI_NIR_30 | 0.50 | 0.45 | 0.20 | 30 |  |
| HET01_DIS_UNI_NIR_00 | 0.50 | 0.45 | 0.20 | 0 |  |
| HET51_DIS_UNI_NIR_00 | 0.50 | 0.45 | 0.20 | 0 |  |
| HET01_DIS_UNI_RED_30 | 0.02 | 0.04 | 0.15 | 30 |  |
| HOM15_DIS_ERE_RED_60 | 0.02 | 0.01 | 0.15 | 60 |  |

# Supplementary 2. Camera calibration

To verify the distortion recovery of the simulated camera, a set of MATLAB 2022b® Computer Vision Tool box (The MathWorks Inc., Natick, MA, USA) built-in images containing a checkerboard with known patch size and patch number captured by a real camera were compared with the checkerboard images captured by a simulated camera in the present framework. These images can be obatined by MATLAB function: “imageDatastore(fullfile(toolboxdir("vision"), "visiondata", "calibration", "mono"))". The verification first calculated the rotation angle and position of the checkerboard in each real camera coordinate and the real camera distortion coefficients by using MATLAB built-in camera calibration models (method of [57]). The obtained information with known checkerboard size and image resolution are input into the present framework to generate checkerboard images under the same settings with corresponding real images. The coefficient of determination betweenchecker square corner positions in real and simulated images are computed for similarity comparison:

(S2)

where and denote the corner positions (either *u* or *v*) in real and simulated images, respectively, is the mean of real positions, and *l* is the total number of positions including both *u* and *v*.

Figure S4 displays a MATLAB built-in checkerboard image alongside simulated checkerboard images with (S4b) and without distortion (S4c). Distorted simulated images have been scaled to accommodate marginal gaps along the edges.

Figure S4. (a) MATLAB built-in real checkerboard images, (b) distorted synthetic checkerboard images, and (c) undistorted synthetic images. For (b), the radial coefficients are -0.3536 and 0.1730, and tangential distortion coefficients are 0.

As for the camera spectral calibration, in Eq. 1 and 2, the integral is a constant . As a simple example case with , , and specified at only 3 discrete wavelengths (), the trapezoidal numerical integration of is:

(S3)

For the integration, the distance between adjacent wavelengths is a constant *w* (if the input spectra have varying wavelength intervals, the camera calibration model will unify the intervals by using linear interpolation). Equation S3 can be reformulated as:

(S4)

which can be converted into a matrix multiplication form:

(S5)

To solve Eq. S5, the model first generates a hyperspectral image (each wavelength has its own image) without an input camera response spectra. The values of the material on the hyperspectral image at all wavelengths, which correspond to , are then extracted. Using the gradient descent method, the camera response spectrum can be recovered by minimizing the cost function .

For the verification of calibrated camera responses, only the RGB bands were tested in this case, as they have the same calibration process as the other bands but are easier to obtain. In the present study, a DGK Color Card (DGK Color Tools, Boston, Massachusetts, USA) with 18 colors was used as the reference material (Fig. 5). The reference reflectivity and transmissivity of each color patch in the board were measured by the PSR+ VIS-NIR field portable spectrometer (Spectral Evolution,Inc., Haverhill, Massachusetts, USA). The color values of each patch in both the real (specified according to online picture) and simulated images were compared by computing the . The calculation is the same as in Eq. S2, where and represent real and simulated color values, respectively, and *l* is the total number of color values, including all RGB channels.

Figure S5 presents the calibrated (color curves) and uncalibrated (black curves) camera response spectra for RGB channels, where the uncalibrated Nikon D700 camera (Nikon Corporation, Tokyo, Japan) response spectrum was sourced from the study of [58] and calibrated response spectrum was based on a real Nikon B500 camera.

Figure S5. Calibrated (colored curves) Nikon B500 camera and uncalibrated (black curves) Nikon D700 camera spectral response for (a) red, (b) green, and (c) blue bands.

# Supplementary 3. Synthetic image examples set-up

Several simple test cases were used to demonstrate potential applications of the modeling framework. Plant canopy geometries of sorghum, strawberry, and bean crops were created using the “Canopy Generator" plug-in in Helios. Default geometric parameters were used. The complex soil geometries for Figs. 8 and 9 were generated using Blender software (Blender Foundation. https://www.blender.org). Specifically, 2D soil texture images were converted into 3D geometries based on pixel values. For some cases, surface reflectance and transmittance spectra for leaves, stems, reproductive organs, and the ground were assigned based on measurements collected in the field using a Spectral Evolution (Haverhill, Massachusetts, USA) PSR+ spectroradiometer and integrating sphere (note that these spectra are included in the default spectral library distributed with Helios). For simplicity, each organ type was assigned constant spectra. For the cases associated with Figs. reffig:Leafopticsbean and 9, spectra were calculated based on the leaf optical model described above.

The camera spectral responses used for Figs. 6, S8, 7, 8 and 9 were calibrated based on a color board image captured by a real Nikon B500 camera under real sun. More specific details regarding camera parameters are given in the figure captions corresponding to each image set.

Generation of synthetic thermal images requires coupling with the Helios Energy Balance Model plug-in and Stomatal Conductance Model plug-in. For simplicity, the default parameter values were used. An example image “annotated" based on leaf net photosynthetic flux is given based on values calculated by the Photosynthesis Model plug-in in Helios. The Farquhar, von Caemmerer and Berry (FvCB) model [46] was used with input parameter values calculated based on the leaf chlorophyll concentration as described in Supplementary 4. The photosynthetically active radiation (PAR) (400-700 nm) flux used for modeling photosynthesis was calculated by using the present ray-tracing model. The net photosynthetic flux in each pixel was determined by mapping the pixel to the corresponding simulated photosynthetic flux of the primitive contained within that pixel.

# Supplementary 4. Net photosynthesis modeling

Leaf net photosynthesis was modeled following [46] based on the implementation described in [25]. The model parameters (maximum carboxylation rate), (maximum electron transport rate), and (dark respiration rate), all at reference temperature of 25C, were specified according to the local leaf chlorophyll concentration.

has an approximately linear relationship with the the chlorophyll concentration :

(S6)

where and are predefined constants and were set to 0.54 cm mol m s and 55.28 mol m s, respectively, based on [59].

is commonly observed to correlate with according to the relationship:

(S7)

where and were set to 1.01 and 0.89 according to [60]. The value for subtracted mitochondrial respiration () was assumed to be 1% of .

# Supplementary 5. Other image examples

Figure S6. Synthetic plant images taken by (a) Nikon D700 camera without any calibration, and by (b) Nikon B500 camera after implementing color calibration and distortion recovery.

Figure S7. Schematic depiction of depth image calculation. The depth is defined as the distance between parallel planes oriented normal to the camera viewing plane that passes through the camera position (camera plane) and passing through the location of the ray-object intersection (object plane).

Figure S8. Synthetic image of sorghum plants under direct sunlight with various zenith (0, 60, 30from top to bottom) and azimuth (0, 90, 270from top to bottom) angles. The source flux varies with the zenith angle, while the scene and the simulated camera remain constant. The camera focal plane distance, HFOV, and diameter of lens are 1.35 m, 30, and 0.02 m, respectively

Figure S9. (a) Background image, (b) Real strawberry RGB image; (c) synthetic strawberry RGB image.

Figure S6: Synthetic plant images taken by uncalibrated and calibrated cameras.

Figure S7: Schematic depiction of depth image calculation. The red point is the target primitive.

Figure S8: Synthetic image of sorghum plants under various sunlight conditions.

Figure S9: Background, real and synthetic images of strawberry plants.

# References

[61] Streher AS, da Silva Torres R, Morellato LPC, and Silva TSF. Accuracy and limitations for spectroscopic prediction of leaf traits in seasonally dry tropical environments. *Remote Sensing of Environment*, 244:111828, 2020.

[62] Weiss M, Jacob F, and Duveiller G. Remote sensing for agricultural applications: A meta-review. *Remote Sensing of Environment*, 236:111402, 2020.

[63] Tattaris M, Reynolds MP, and Chapman SC. A direct comparison of remote sensing approaches for high-throughput phenotyping in plant breeding. *Frontiers in Plant Science*, 7:1131, 2016.

[64] Longchamps L, Tisseyre B, Taylor J, Sagoo L, Momin A, Fountas S, Manfrini L, Ampatzidis Y, Schueller JK, and Khosla R. Yield sensing technologies for perennial and annual horticultural crops: a review. *Precision Agriculture*, pages 1–42, 2022.

[65] Singh A, Ganapathysubramanian B, Singh AK, and Sarkar S. Machine learning for high-throughput stress phenotyping in plants. *Trends in Plant Science*, 21(2):110–124, 2016.

[66] Mwinuka PR, Mourice SK, Mbungu WB, Mbilinyi BP, Tumbo SD, and Schmitter P. UAV-based multispectral vegetation indices for assessing the interactive effects of water and nitrogen in irrigated horticultural crops production under tropical sub-humid conditions: A case of African eggplant. *Agricultural Water Management*, 266:107516, 2022.

[67] Lan Y, Huang Z, Deng X, Zhu Z, Huang H, Zheng Z, Lian B, Zeng G, and Tong Z. Comparison of machine learning methods for citrus greening detection on UAV multispectral images. *Computers and Electronics in Agriculture*, 171:105234, 2020.

[68] Jay S, Baret F, Dutartre D, Malatesta G, Héno S, Comar A, Weiss M, and Maupas F. Exploiting the centimeter resolution of uav multispectral imagery to improve remote-sensing estimates of canopy structure and biochemistry in sugar beet crops. *Remote Sensing of Environment*, 231:110898, 2019.

[69] Yu N, Li L, Schmitz N, Tian LF, Greenberg JA, and Diers BW. Development of methods to improve soybean yield estimation and predict plant maturity with an unmanned aerial vehicle based platform. *Remote Sensing of Environment*, 187:91–101, 2016.

[70] Maimaitijiang M, Sagan V, Sidike P, Hartling S, Esposito F, and Fritschi FB. Soybean yield prediction from UAV using multimodal data fusion and deep learning. *Remote Sensing of Environment*, 237:111599, 2020.

[71] Roth L, Barendregt C, Bétrix CA, Hund A, and Walter A. High-throughput field phenotyping of soybean: Spotting an ideotype. *Remote Sensing of Environment*, 269:112797, 2022.

[72] Shafiekhani A, Fritschi FB, and DeSouza GN. Visnd: A visualization tool for multidimensional model of canopy. In *Proceedings of the IEEE/CVF Conference on Computer Vision and Pattern Recognition Workshops*, pages 0–0, 2019.

[73] Jeffrey A Cruz, Xi Yin, Xiaoming Liu, Saif M Imran, Daniel D Morris, David M Kramer, and Jin Chen. Multi-modality imagery database for plant phenotyping. *Machine Vision and Applications*, 27:735–749, 2016.

[74] Xie P, Du R, Ma Z, and Cen H. Generating 3D multispectral point clouds of plants with fusion of snapshot spectral and RGB-D images. *Plant Phenomics*, 5:0040, 2023.

[75] Uchiyama H, Sakurai S, Mishima M, Arita D, Okayasu T, Shimada A, and Taniguchi R. An Easy-to-setup 3D phenotyping platform for KOMATSUNA dataset. In *Proceedings of the IEEE International Conference on Computer Vision Workshops*, pages 2038–2045, 2017.

[76] Strahler AH. Vegetation canopy reflectance modeling–recent developments and remote sensing perspectives. *Remote Sensing Reviews*, 15(1-4):179–194, 1997.

[77] Widlowski JL, Pinty B, Lopatka M, Atzberger C, Buzica D, Chelle M, Disney M, Gastellu-Etchegorry JP, Gerboles M, Gobron N, Grau E, Huang H, Kallel A, Kobayashi H, Lewis PE, Qin W, Schlerf M, Stuckens J, and Xie D. The fourth radiation transfer model intercomparison (RAMI-IV): Proficiency testing of canopy reflectance models with ISO-13528. *Journal of Geophysical Research*, 118:6869–6890, 2013.

[78] Jacquemoud S. Inversion of the PROSPECT+ SAIL canopy reflectance model from AVIRIS equivalent spectra: theoretical study. *Remote Sensing of Environment*, 44(2-3):281–292, 1993.

[79] Bailey BN. A reverse ray-tracing method for modelling the net radiative flux in leaf-resolving plant canopy simulations. *Ecological Modelling*, 368:233–245, 2018.

[80] Qi J, Xie D, Yin T, Yan G, Gastellu-Etchegorry JP, Li L, Zhang W, Mu X, and Norford LK. LESS: LargE-Scale remote sensing data and image simulation framework over heterogeneous 3D scenes. *Remote Sensing of Environment*, 221:695–706, 2019.

[81] Pound MP, Atkinson JA, Wells DM, Pridmore TP, and French A P. Deep learning for multi-task plant phenotyping. In *2017 IEEE International Conference on Computer Vision Workshops (ICCVW)*, pages 2055–2063, 2017.

[82] Joshi A, Guevara D, and Earles M. Standardizing and centralizing datasets to enable efficient training of agricultural deep learning models. *arXiv preprint arXiv:2208.02707*, 2022.

[83] Feng X, Zhan Y, Wang Q, Yang X, Yu C, Wang H, Tang ZY, Jiang D, Peng C, and He Y. Hyperspectral imaging combined with machine learning as a tool to obtain high-throughput plant salt-stress phenotyping. *The Plant Journal*, 101(6):1448–1461, 2020.

[84] Rehman TU, Ma D, Wang L, Zhang L, and Jin J. Predictive spectral analysis using an end-to-end deep model from hyperspectral images for high-throughput plant phenotyping. *Computers and Electronics in Agriculture*, 177:105713, 2020.

[85] Bailey BN. Helios: A scalable 3D plant and environmental biophysical modeling framework. *Frontiers in Plant Science*, 10:1185, 2019.

[86] Fei Z, Olenskyj AG, Bailey BN, and Earles M. Enlisting 3D crop models and GANs for more data efficient and generalizable fruit detection. In *Proceedings of the IEEE/CVF International Conference on Computer Vision*, pages 1269–1277, 2021.

[87] Wang Y and Gastellu-Etchegorry JP. Accurate and fast simulation of remote sensing images at top of atmosphere with DART-Lux. *Remote Sensing of Environment*, 256:112311, 2021.

[88] Féret JB, Gitelson AA, Noble SD, and Jacquemoud S. PROSPECT-D: Towards modeling leaf optical properties through a complete lifecycle. *Remote Sensing of Environment*, 193:204–215, 2017.

[89] Féret JB, Berger K, De Boissieu F, and Malenovskỳ Z. PROSPECT-PRO for estimating content of nitrogen-containing leaf proteins and other carbon-based constituents. *Remote Sensing of Environment*, 252:112173, 2021.

[90] Modest MF. *Radiative Heat Transfer*. Academic Press, Waltham, MA, third edition, 2013. 904 pp.

[91] Wagner EP, Merz J, and Townsend PA. Ecological spectral information system: An open spectral library. In *AGU Fall Meeting Abstracts*, volume 2018, pages B41L–2878, 2018.

[92] Suffern K. *Ray Tracing from the Ground up*. CRC Press, 2016.

[93] Bailey BN, Stoll R, Pardyjak ER, and Miller NE. A new three-dimensional energy balance model for complex plant canopy geometries: Model development and improved validation strategies. *Agricultural and Forest Meteorology*, 218:146–160, 2016.

[94] Widlowski JL, Robustelli M, Disney M, Gastellu-Etchegorry JP, Lavergne T, Lewis P, North PRJ, Pinty B, Thompson R, and Verstraete MM. The RAMI On-line Model Checker (ROMC): A web-based benchmarking facility for canopy reflectance models. *Remote Sensing of Environment*, 112(3):1144–1150, 2008.

[95] Widlowski JL, Taberner M, Pinty B, Bruniquel-Pinel V, Disney M, Fernandes R, Gastellu-Etchegorry JP, Gobron N, Kuusk A, Lavergne T, et al. Third Radiation Transfer Model Intercomparison (RAMI) exercise: Documenting progress in canopy reflectance models. *Journal of Geophysical Research: Atmospheres*, 112(D9), 2007.

[96] Widlowski JL, Mio C, Disney M, Adams J, Andredakis I, Atzberger C, Brennan J, Busetto L, Chelle M, Ceccherini G, and Colombo R. The fourth phase of the radiative transfer model intercomparison (RAMI) exercise: Actual canopy scenarios and conformity testing. *Remote Sensing of Environment*, 169:418–437, 2015.

[97] Chéné Y, Rousseau D, Lucidarme P, Bertheloot J, Caffier V, Morel P, Belin É, and Chapeau-Blondeau F. On the use of depth camera for 3D phenotyping of entire plants. *Computers and Electronics in Agriculture*, 82:122–127, 2012.

[98] Lu Y, Wang Y, Chen Z, Khan A, Salvaggio C, and Lu G. 3D plant root system reconstruction based on fusion of deep structure-from-motion and IMU. *Multimedia Tools and Applications*, 80:17315–17331, 2021.

[99] Zhou Y, Rupnik E, Meynard C, Thom C, and Pierrot-Deseilligny M. Simulation and analysis of photogrammetric UAV image blocks–Influence of camera calibration error. *Remote Sensing*, 12(1):22, 2019.

[100] Maes WH and Steppe K. Perspectives for remote sensing with unmanned aerial vehicles in precision agriculture. *Trends in Plant Science*, 24(2):152–164, 2019.

[101] Heikkila J and Silvén O. A four-step camera calibration procedure with implicit image correction. In *Proceedings of IEEE Computer Society Conference on Computer Vision and Pattern Recognition*, pages 1106–1112. IEEE, 1997.

[102] Jacquemoud S and Baret F. PROSPECT: A model of leaf optical properties spectra. *Remote Sensing of Environment*, 34(2):75–91, 1990.

[103] Allen WA, Gausman HW, and Richardson AJ. Interaction of isotropic light with a compact plant leaf. *Josa*, 59(10):1376–1379, 1969.

[104] Jocher G, Chaurasia A, Stoken A, Borovec J, NanoCode012, Kwon Y, Michael K, TaoXie, Fang J, imyhxy, Lorna, Yifu Z, Wong C, V Abhiram, Montes D, Wang Z, Fati C, Nadar J, Laughing, et al. ultralytics/yolov5: v7.0 - yolov5 sota realtime instance segmentation, 2022.

[105] Weng S, Yu S, Guo B, Tang P, and Liang D. Non-destructive detection of strawberry quality using multi-features of hyperspectral imaging and multivariate methods. *Sensors*, 20(11):3074, 2020.

[106] Farquhar GD, Schulze E−D, and Küppers M. Responses to humidity by stomata of *Nicotiana glauca* L. and *Corylus avellana* L. are consistent with the optimization of carbon dioxide uptake with respect to water loss. *Functional Plant Biology*, 7:315–327, 1980.

[107] de León MA and Bailey BN. A 3D model for simulating spatial and temporal fluctuations in grape berry temperature. *Agricultural and Forest Meteorology*, 306:108431, 2021.

[108] Mayanja IK, Diepenbrock CH, Vadez V, Lei T, and Bailey BN. Practical considerations and limitations of using leaf and canopy temperature measurements as a stomatal conductance proxy: sensitivity across environmental conditions, scale, and sample size. *Plant Phenomics*, 6:0169, 2024.

[109] Hartley ZKJ and French AP. Domain adaptation of synthetic images for wheat head detection. *Plants*, 10(12):2633, 2021.

[110] Chen Q, Zheng B, Chenu K, Hu P, and Chapman SC. Unsupervised plot-scale lai phenotyping via uav-based imaging, modelling, and machine learning. *Plant Phenomics*, 2022, 2022.

[111] Xia C, Wang L, Chung BK, and Lee JM. In situ 3D segmentation of individual plant leaves using a RGB-D camera for agricultural automation. *Sensors*, 15(8):20463–20479, 2015.

[112] An N, Welch SM, Markelz RJC, Baker RL, Palmer CM, Ta J, Maloof JN, and Weinig C. Quantifying time-series of leaf morphology using 2D and 3D photogrammetry methods for high-throughput plant phenotyping. *Computers and Electronics in Agriculture*, 135:222–232, 2017.

[113] Wang H, Duan Y, Shi Y, Kato Y, Ninomiya S, and Guo W. EasyIDP: A Python package for intermediate data processing in UAV-based plant phenotyping. *Remote Sensing*, 13(13):2622, 2021.

[114] Gehan MA, Fahlgren N, Abbasi A, Berry JC, Callen ST, Chavez L, Doust AN, Feldman MJ, Gilbert KB, Hodge JG, et al. PlantCV v2: Image analysis software for high-throughput plant phenotyping. *PeerJ*, 5:e4088, 2017.

[115] ElManawy AI, Sun D, Abdalla A, Zhu Y, and Cen H. HSI-PP: A flexible open-source software for hyperspectral imaging-based plant phenotyping. *Computers and Electronics in Agriculture*, 200:107248, 2022.

[116] Reynolds D, Ball J, Bauer A, Davey R, Griffiths S, and Zhou J. CropSight: A scalable and open-source information management system for distributed plant phenotyping and IoT-based crop management. *Gigascience*, 8(3):giz009, 2019.

[117] Zhang Z. A flexible new technique for camera calibration. *IEEE Transactions on Pattern Analysis and Machine Intelligence*, 22(11):1330–1334, 2000.

[118] Mauer C and Wueller D. Measuring the spectral response with a set of interference filters. In *Digital photography V*, volume 7250, pages 235–244. SPIE, 2009.

[119] Qian X, Liu L, Croft H, and Chen J. Relationship between leaf maximum carboxylation rate and chlorophyll content preserved across 13 species. *Journal of Geophysical Research: Biogeosciences*, 126(2):e2020JG006076, 2021.

[120] Walker AP, Beckerman AP, Gu L, Kattge J, Cernusak LA, Domingues TF, Scales JC, Wohlfahrt G, Wullschleger SD, and Woodward FI. The relationship of leaf photosynthetic traits–Vcmax and Jmax–to leaf nitrogen, leaf phosphorus, and specific leaf area: A meta-analysis and modeling study. *Ecology and Evolution*, 4(16):3218–3235, 2014.
