# Supplementary material for: Simulation of Automatically Annotated Visible and Multi-/Hyperspectral Images Using the Helios 3D Plant and Radiative Transfer Modeling Framework
Source: Plant Phenomics. 2024 May 30;6:0189. doi: 10.34133/plantphenomics.0189 (PMC11136674; doi:10.34133/plantphenomics.0189)
Supplement: Supplementary 1 — Sections S1 to S6 Figs. S1 to S9 Tables S1 and S2 References [54–60] [file plantphenomics.0189.f1.zip › Supplementary new.pdf]

Simulation of automatically annotated visible and multi/ hyperspectral images using the Helios 3D plant and radiative transfer modeling framework

Supplementary 1 ROMC settings and verification results

Two measurements were used for verifying the radiation with single scattering received by the simulated camera (Table S1): *brfpp_uc_sgl* and *brfpp_co_sgl*, which measure the Bi-directional Reflectance Factor (BRF) of soil and canopy in the principal plane, respectively. BRF is defined as the radiant flux exiting from a target area in a particular direction, passing through a planar reference surface, and normalized by the equivalent quantity leaving from an infinitely large Lambertian background via the same reference surface and for the same directions of illumination and reflection. A Lambertian background represents a perfectly reflecting isotropic surface according to the ROMC definition. The reference surface, situated perpendicularly to the normal of the underlying surface, has defined lateral dimensions and elevation above the target area. The principle plane implies that when the sun azimuth angle is 0°, the viewing azimuth angle corresponds to 180°. The image size for the simulated cameras changed with zenith angle to ensure that only radiation fluxes that passed through the reference plane were collected. The distance between the canopies and the simulated camera was set to a very large value (100,000 m) to guarantee that the reflected radiation fluxes were nearly parallel and very close to the prescribed viewing zenith angle.

Another two measurements *fabs* and *brfop* (Table S1) were employed to measure the percentage of radiation entering the canopy via the reference plane that has been absorbed by the leaves excluding the soil, and BRF with multiple scattering in the cross plane, respectively. The cross plane is oriented perpendicularly to the principal plane, representing a 90° viewing azimuth angle when the sun azimuth angle is 0°. All experiment IDs are listed in Table S1. The ROMC website provides comprehensive information on scene details, including geometries, scene extent, radiative properties, and sun and viewing directions, which can be easily accessed by searching for these experiment IDs.

The SKILL score is the indicator of the model performance for all of these four measurements, which can be computed as:

$$\mathrm{SKILL} = \frac{(1+R)^4}{(\frac{\bar{x}_{usr}}{\bar{x}_{ref}} + \frac{\bar{x}_{ref}}{\bar{x}_{usr}})^2(\frac{\sigma_{usr}}{\sigma_{ref}} + \frac{\sigma_{ref}}{\sigma_{usr}})^2}, \quad \text{(S1)}$$

where R denotes the Pearson correlation coefficient, $\bar{x}_{usr}$ and $\bar{x}_{ref}$ are the mean values of results obtained by present framework and the ROMC reference results, respectively, and σ_{usr} and σ_{ref} indicate the standard deviation of results. Various experiments containing both heterogeneous and homogeneous canopies under different sun zenith angles were randomly selected for the verification test.

Figure S1 presents the BRF simulated results (red curves) against the ROMC reference results of the *brop* case. Table S2 provides the ROMC experiment IDs employed for verifying the present ray-tracing model. For *fabs* and *brfop*, the radiation flux and scattering depth (iteration) were assigned

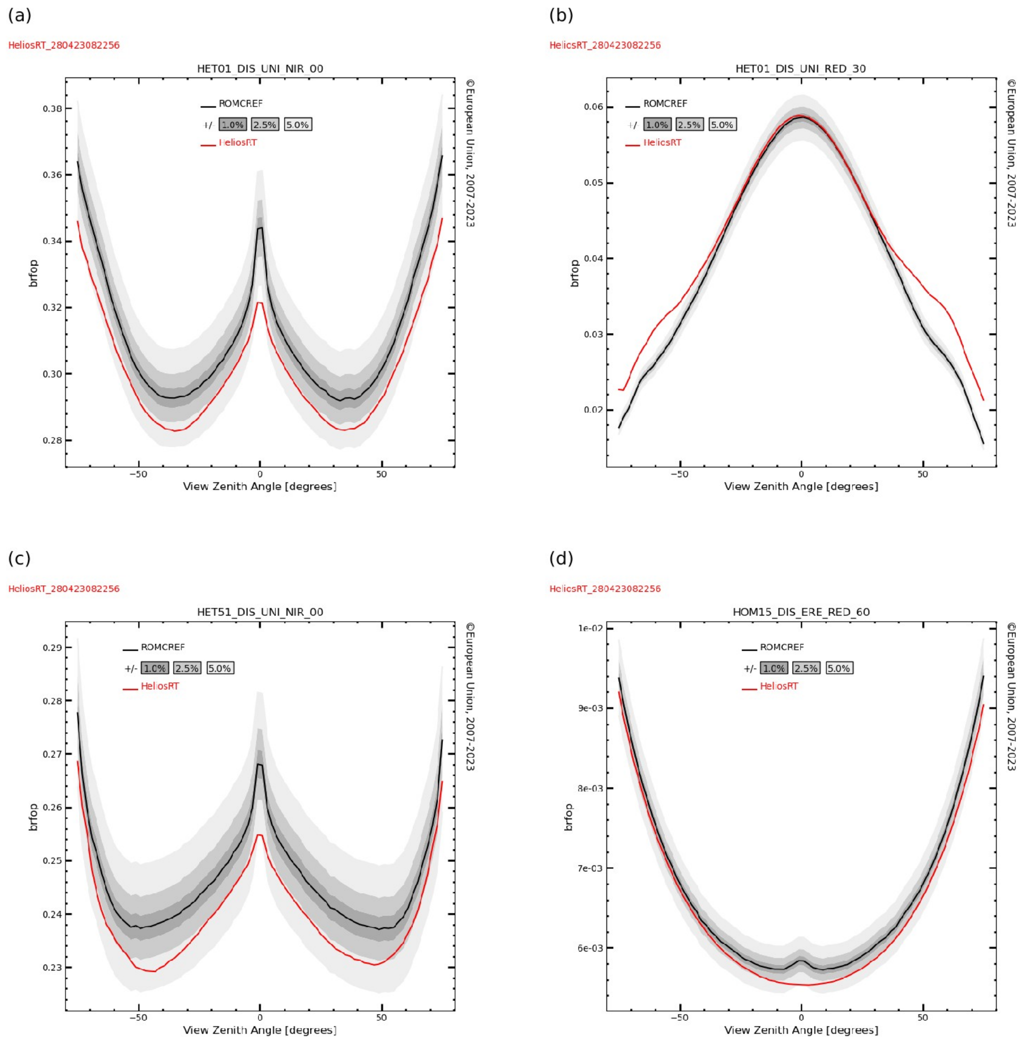

Figure S1: ROMC summary result of *brfop* measurement

Table S1: Selected ROMC measurement IDs and experiments for verification

Measurement	*brfpp_uc_sgl* and *brfpp_co_sgl*	*brfop* and *fabs*
Experiment1	HET21_DIS_UNI_RED_00	HOM15_DIS_ERE_RED_60
Experiment2	HET11_DIS_UNI_RED_30	HET01_DIS_UNI_RED_30
Experiment3	HET21_DIS_UNI_NIR_30	HET51_DIS_UNI_NIR_00
Experiment4	HET01_DIS_UNI_NIR_00	HET01_DIS_UNI_NIR_00

Table S2: ROMC scene settings for verification tests

Experiment1	Leaf reflectance	Leaf transmittance	Soil reflectance	Solar Zenith Angle [deg]	Domain size [m^3]
HET21_DIS_UNI_RED_00	0.02	0.01	0.15	0	$100 \times 100 \times 25$
HET11_DIS_UNI_RED_30	0.02	0.01	0.15	30	$100 \times 100 \times 25$
HET21_DIS_UNI_NIR_30	0.50	0.45	0.20	30	$100 \times 100 \times 25$
HET01_DIS_UNI_NIR_00	0.50	0.45	0.20	0	$100 \times 100 \times 25$
HET51_DIS_UNI_NIR_00	0.50	0.45	0.20	0	$100 \times 100 \times 25$
HET01_DIS_UNI_RED_30	0.02	0.04	0.15	30	$100 \times 100 \times 25$
HOM15_DIS_ERE_RED_60	0.02	0.01	0.15	60	$25 \times 25 \times 1$

values of 10 W/m^2 and 20, respectively. For each element, the number of direct rays was set to 100, while the number of diffuse rays was set to 1,000, both of which are default settings in Helios. For the measurements *brfpp_uc_sgl*, *brfpp_co_sgl*, and *brfop*, 2-degree intervals of the view zenith angle were set, covering a range from -75 degrees to 75 degrees in the target plane. The camera resolution was set to 1000×1000, and a total of 100 rays were launched from each pixel on the camera image plane. The test scenes for these three measurements were duplicated to the left, right, up, and down of the target test scene used for observing. This was done to ensure radiative energy conservation in the observation plane. As a result, there are five replicated test scenes for each of these three measurements. Figure S2 shows BRF curves of experiment HET01_DIS_UNI_NIR_00 measured in the ROMC case *brfop* by using 5, 10, and 20 scattering iterations.

For the actual scene "Wellington Citrus Orchard" with experiment ID of HET14_WCO_090_B01_20 from RAMIV IV [35], Fig. S3 shows the BRF (*brfpp*) curves obtained by our ray-tracing model and the bench mark models provided by the RAMI IV website. The *brfpp* is similar to the *brfop*, while it sets 0° viewing azimuth angle when the sun azimuth angle is 0°. Detailed information is available on the RAMI IV website by searching the experiment ID: HET14_WCO_090_B01_20.

Supplementary 2 Camera calibration

To verify the distortion recovery of the simulated camera, a set of MATLAB® 2022b Computer Vision Tool box (The MathWorks Inc., Natick, MA, USA) built-in images containing a checkerboard with known patch size and patch number captured by a real camera were compared with the checkerboard images captured by a simulated camera in the present framework. These images can be obatined by MATLAB function: "imageDatastore(fullfile(toolboxdir("vision"), "visiondata", "calibration", "mono"))". The verification first calculated the rotation angle and position of the checkerboard in each real camera coordinate and the real camera distortion coefficients by using

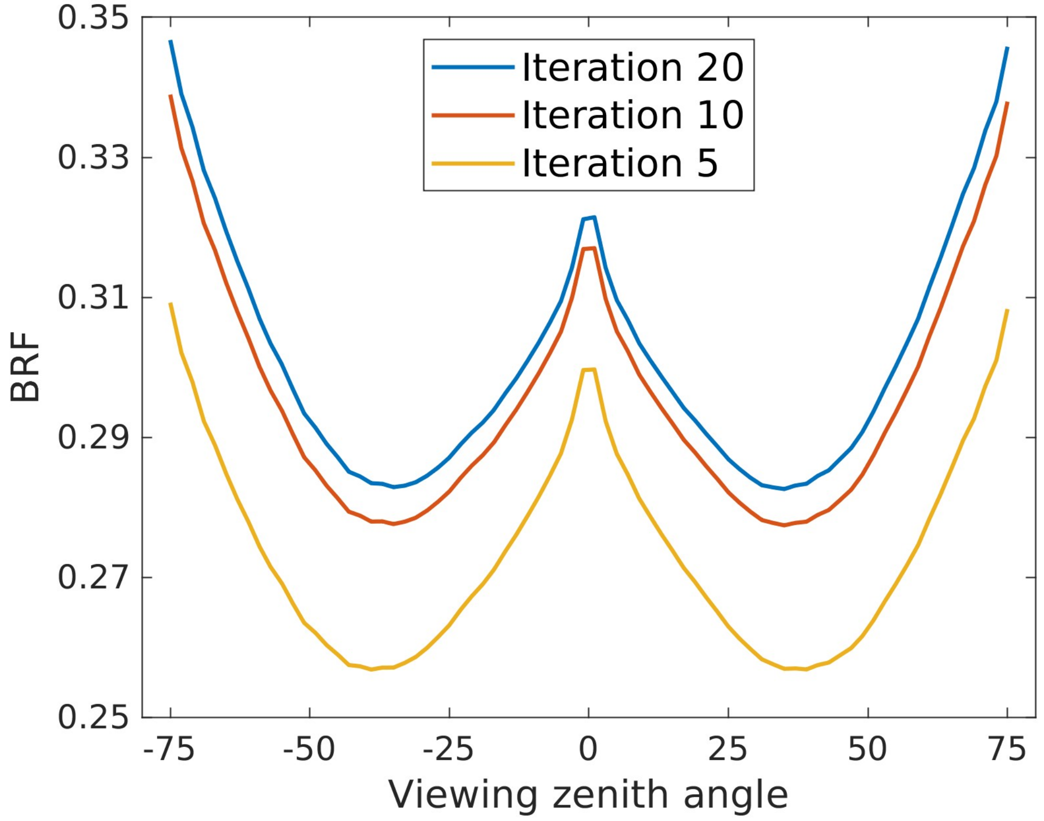

Figure S2: The Bi-Directional Reflectance Factor (BRF) curve of experiment HET01_DIS_UNI_NIR_00 measured in the ROMC case *brfop* by using 5, 10, and 20 scattering iterations.

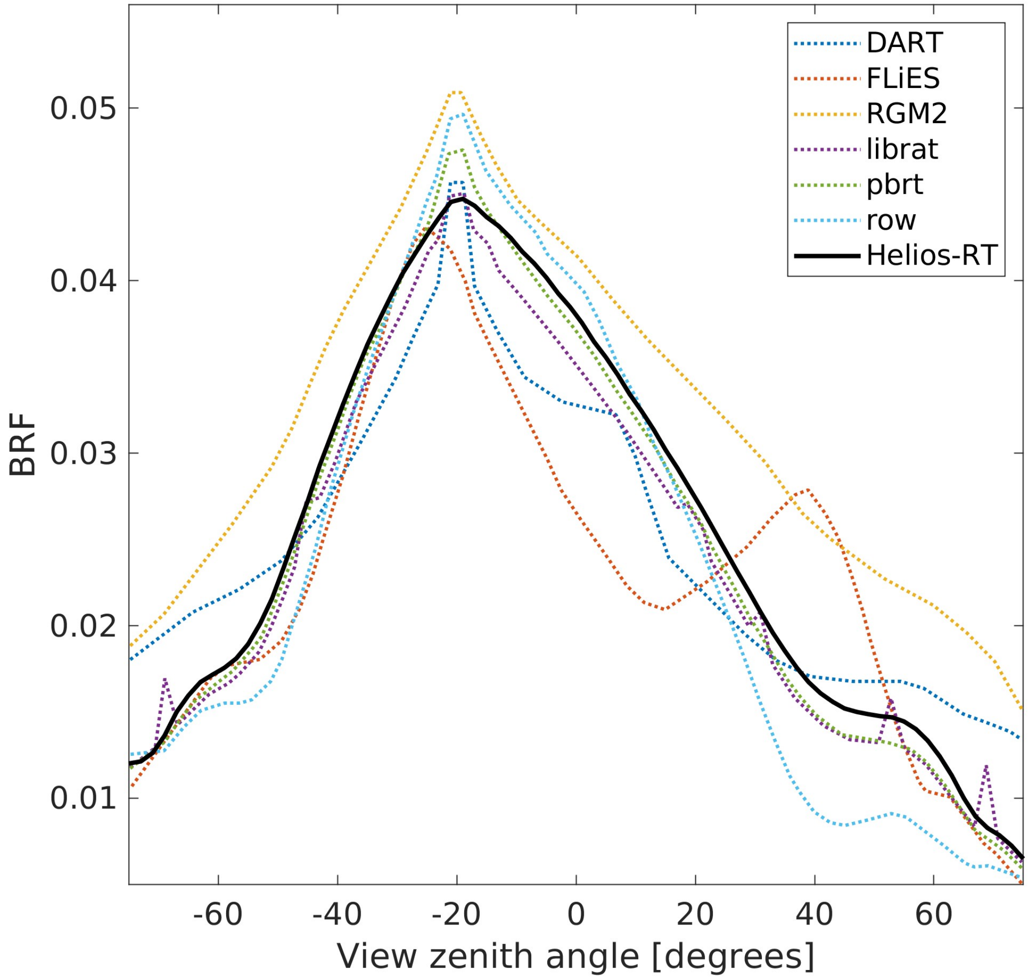

Figure S3: The BRF (*brfpp*) curves of the actual scene "Wellington Citrus Orchard" obtained by RAMI IV bench mark models and the present ray-tracing model (Helios-RT). The details and reference of these benchmark models can be found in [35].

MATLAB built-in camera calibration models (method of [54]). The obtained information with known checkerboard size and image resolution are input into the present framework to generate checkerboard images under the same settings with corresponding real images. The coefficient of determination R^2 between checker square corner positions in real and simulated images are computed for similarity comparison:

$$R^2 = 1 - \frac{\sum_{i=1}^{l}(y_{real,i} - y_{sim,i})^2}{\sum_{i=1}^{l}(y_{real,i} - \bar{y}_{real})^2} \tag{S2}$$

where $y_{real,i}$ and $y_{sim,i}$ denote the corner positions (either u or v) in real and simulated images, respectively, $\bar{y}_{real}$ is the mean of real positions, and l is the total number of positions including both u and v.

Figure S4 displays a MATLAB built-in checkerboard image alongside simulated checkerboard images with (Fig. S4b) and without distortion (Fig. S4c). Distorted simulated images have been scaled to accommodate marginal gaps along the edges.

As for the camera spectral calibration, in Eq. 1 and 2, the integral $\int_{\lambda_{min}}^{\lambda_{max}} S_\lambda \, d\lambda$ is a constant μ. As a simple example case with ρ_λ, C_λ, and S_λ specified at only 3 discrete wavelengths ($\lambda_1 < \lambda_2 < \lambda_3$), the trapezoidal numerical integration of $\int_{\lambda_1}^{\lambda_3} \rho_\lambda \, C_\lambda \, S_\lambda \, d\lambda$ is:

$$\mu \, m_{sim} = 0.5((\lambda_2 - \lambda_1)(C_1 \, \rho_1 \, S_1 + C_2 \, \rho_2 \, S_2) + (\lambda_3 - \lambda_2)(C_2 \, \rho_2 \, S_2 + C_3 \, \rho_3 \, S_3)). \tag{S3}$$

For the integration, the distance between adjacent wavelengths is a constant w (if the input spectra have varying wavelength intervals, the camera calibration model will unify the intervals by using linear interpolation). Equation S3 can be reformulated as:

$$\mu \, m_{sim} = 0.5w(C_1 \, \rho_1 \, S_1 + 2C_2 \, \rho_2 \, S_2 + C_3 \, \rho_3 \, S_3), \tag{S4}$$

which can be converted into a matrix multiplication form:

$$2m_{sim} = [C_1, C_2, C_3] * [\rho_1 \, S_1, 2\rho_2 \, S_2, \rho_3 \, S_3]^T \, w/\mu, \text{ subject to } C_\lambda > 0. \tag{S5}$$

To solve Eq. S5, the model first generates a hyperspectral image (each wavelength λ has its own image) without an input camera response spectra. The values of the material on the hyperspectral image at all wavelengths, which correspond to $\rho \, I \mu \, w$, are then extracted. Using the gradient descent method, the camera response spectrum $[C_1, C_2, C_3]$ can be recovered by minimizing the cost function $0.5(m_{real} - m_{sim})^2$.

For the verification of calibrated camera responses, only the RGB bands were tested in this case, as they have the same calibration process as the other bands but are easier to obtain. In the present study, a DGK Color Card (DGK Color Tools, Boston, Massachusetts, USA) with 18 colors was used as the reference material (Fig. 5). The reference reflectivity and transmissivity of each color patch in the board were measured by the PSR+ VIS-NIR field portable spectrometer (Spectral Evolution, Inc., Haverhill, Massachusetts, USA). The color values of each patch in both the real

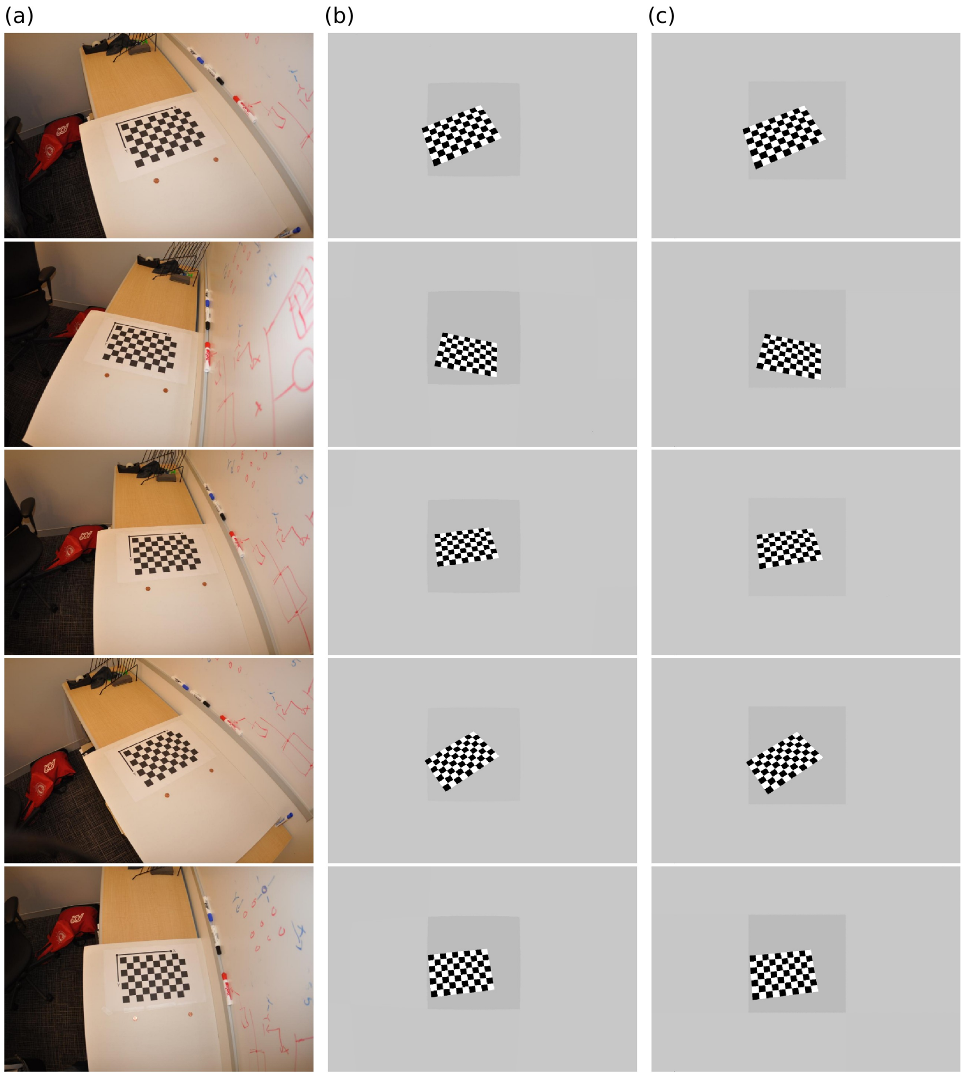

Figure S4: (a) MATLAB built-in real checkerboard images, (b) distorted synthetic checkerboard images, and (c) undistorted synthetic images. For (b), the radial coefficients are -0.3536 and 0.1730, and tangential distortion coefficients are 0.

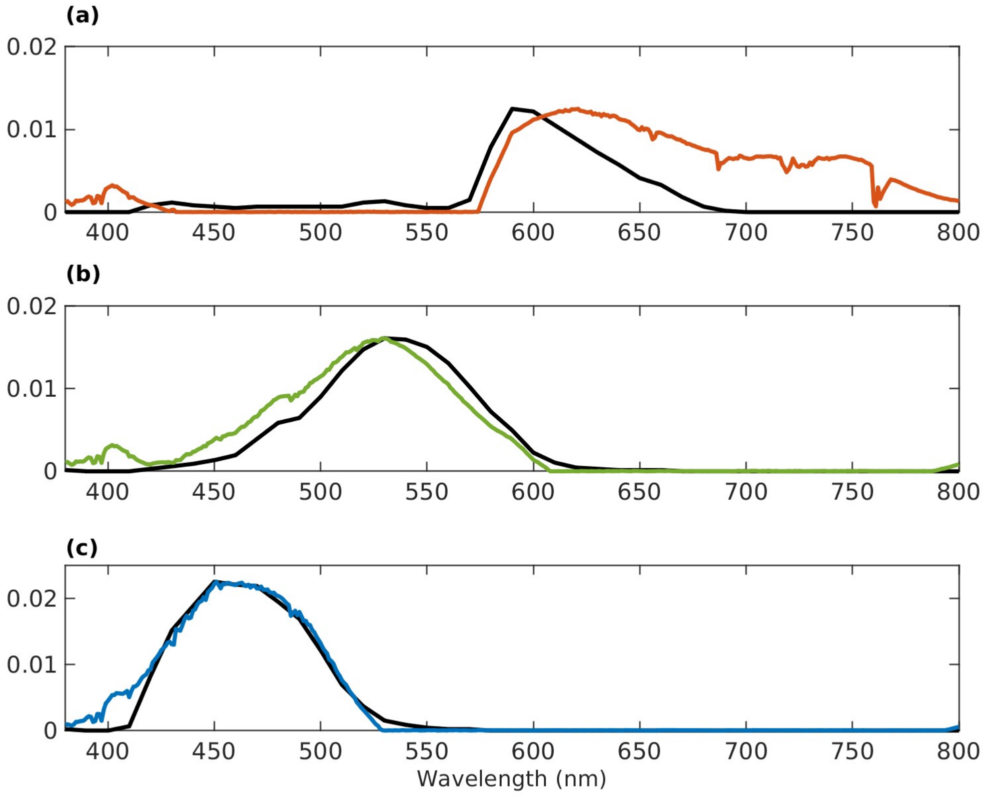

Figure S5: Calibrated (colored curves) Nikon B500 camera and uncalibrated (black curves) Nikon D700 camera spectral response for (a) red, (b) green, and (c) blue bands.

(specified according to online picture) and simulated images were compared by computing the R^2. The calculation is the same as in Eq. S2, where $y_{real,i}$ and $y_{sim,i}$ represent real and simulated color values, respectively, and l is the total number of color values, including all RGB channels.

Figure S5 presents the calibrated (color curves) and uncalibrated (black curves) camera response spectra for RGB channels, where the uncalibrated Nikon D700 camera (Nikon Corporation, Tokyo, Japan) response spectrum was sourced from the study of [55] and calibrated response spectrum was based on a real Nikon B500 camera.

Supplementary 3 Synthetic image examples set-up

Several simple test cases were used to demonstrate potential applications of the modeling framework. Plant canopy geometries of sorghum, strawberry, and bean crops were created using the “Canopy Generator” plug-in in Helios. Default geometric parameters were used. The complex soil geometries for Figs. 8 and 9 were generated using Blender software (Blender Foundation. `https://www.blender.org`). Specifically, 2D soil texture images were converted into 3D geometries based on pixel

values. For some cases, surface reflectance and transmittance spectra for leaves, stems, reproductive organs, and the ground were assigned based on measurements collected in the field using a Spectral Evolution (Haverhill, Massachusetts, USA) PSR+ spectroradiometer and integrating sphere (note that these spectra are included in the default spectral library distributed with Helios). For simplicity, each organ type was assigned constant spectra. For the cases associated with Figs. 8 and 9, spectra were calculated based on the leaf optical model described above.

The camera spectral responses used for Figs. 6, S8, 7, 8 and 9 were calibrated based on a color board image captured by a real Nikon B500 camera under real sun. More specific details regarding camera parameters are given in the figure captions corresponding to each image set.

Generation of synthetic thermal images requires coupling with the Helios Energy Balance Model plug-in and Stomatal Conductance Model plug-in. For simplicity, the default parameter values were used. An example image "annotated" based on leaf net photosynthetic flux is given based on values calculated by the Photosynthesis Model plug-in in Helios. The Farquhar, von Caemmerer and Berry (FvCB) model [43] was used with input parameter values calculated based on the leaf chlorophyll concentration as described in Supplementary 4. The photosynthetically active radiation (PAR) (400-700 nm) flux used for modeling photosynthesis was calculated by using the present ray-tracing model. The net photosynthetic flux in each pixel was determined by mapping the pixel to the corresponding simulated photosynthetic flux of the primitive contained within that pixel.

Supplementary 4 Net photosynthesis modeling

Leaf net photosynthesis was modeled following [43] based on the implementation described in [25]. The model parameters V_{cmax25} (maximum carboxylation rate), J_{max25} (maximum electron transport rate), and R_{d25} (dark respiration rate), all at reference temperature of 25°C, were specified according to the local leaf chlorophyll concentration.

V_{cmax25} has an approximately linear relationship with the the chlorophyll concentration W_{chl}:

$$V_{cmax25} = a_{vc} W_{chl} + b_{vc}, \tag{S6}$$

where a_{vc} and b_{vc} are predefined constants and were set to 0.54 cm^2 μmol m^{-2} μg^{-1} s^{-1} and 55.28 μmol m^{-2} s^{-1}, respectively, based on [56].

J_{max25} is commonly observed to correlate with V_{cmax25} according to the relationship:

$$\ln\left(J_{max25}\right) = a_{jv} \ln\left(V_{cmax25}\right) + b_{jv}, \tag{S7}$$

where b_{jv} and a_{jv} were set to 1.01 and 0.89 according to [57]. The value for subtracted mitochondrial respiration (R_{d25}) was assumed to be 1% of V_{cmax25}.

Supplementary 5 Other image examples

Figure S6: Synthetic plant images taken by uncalibrated and calibrated cameras.

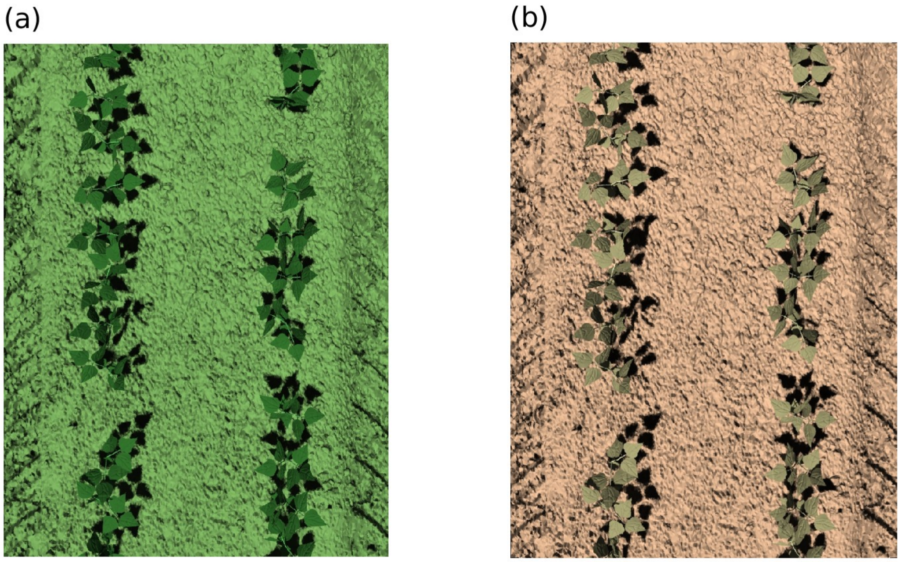

Figure S6: Synthetic plant images taken by (a) Nikon D700 camera without any calibration, and by (b) Nikon B500 camera after implementing color calibration and distortion recovery.

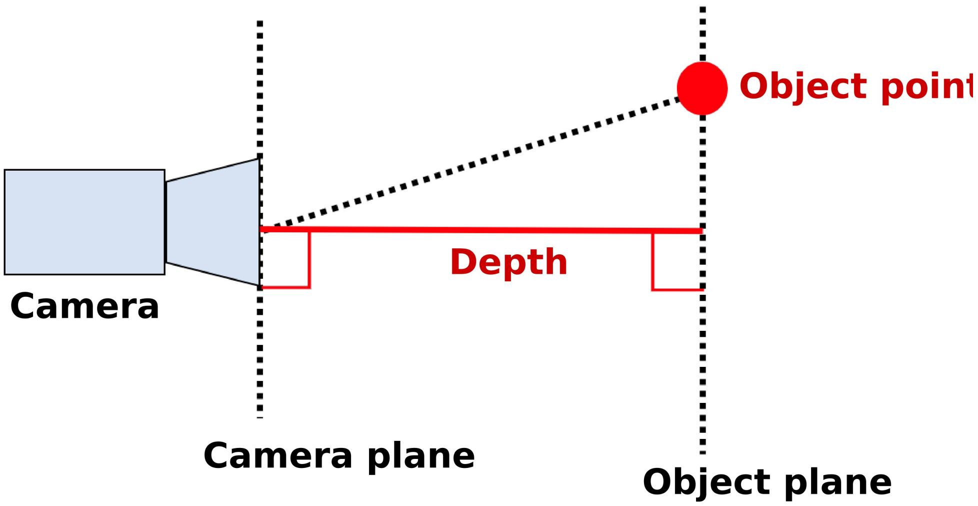

Figure S7: Schematic depiction of depth image calculation. The depth is defined as the distance between parallel planes oriented normal to the camera viewing plane that passes through the camera position (camera plane) and passing through the location of the ray-object intersection (object plane).

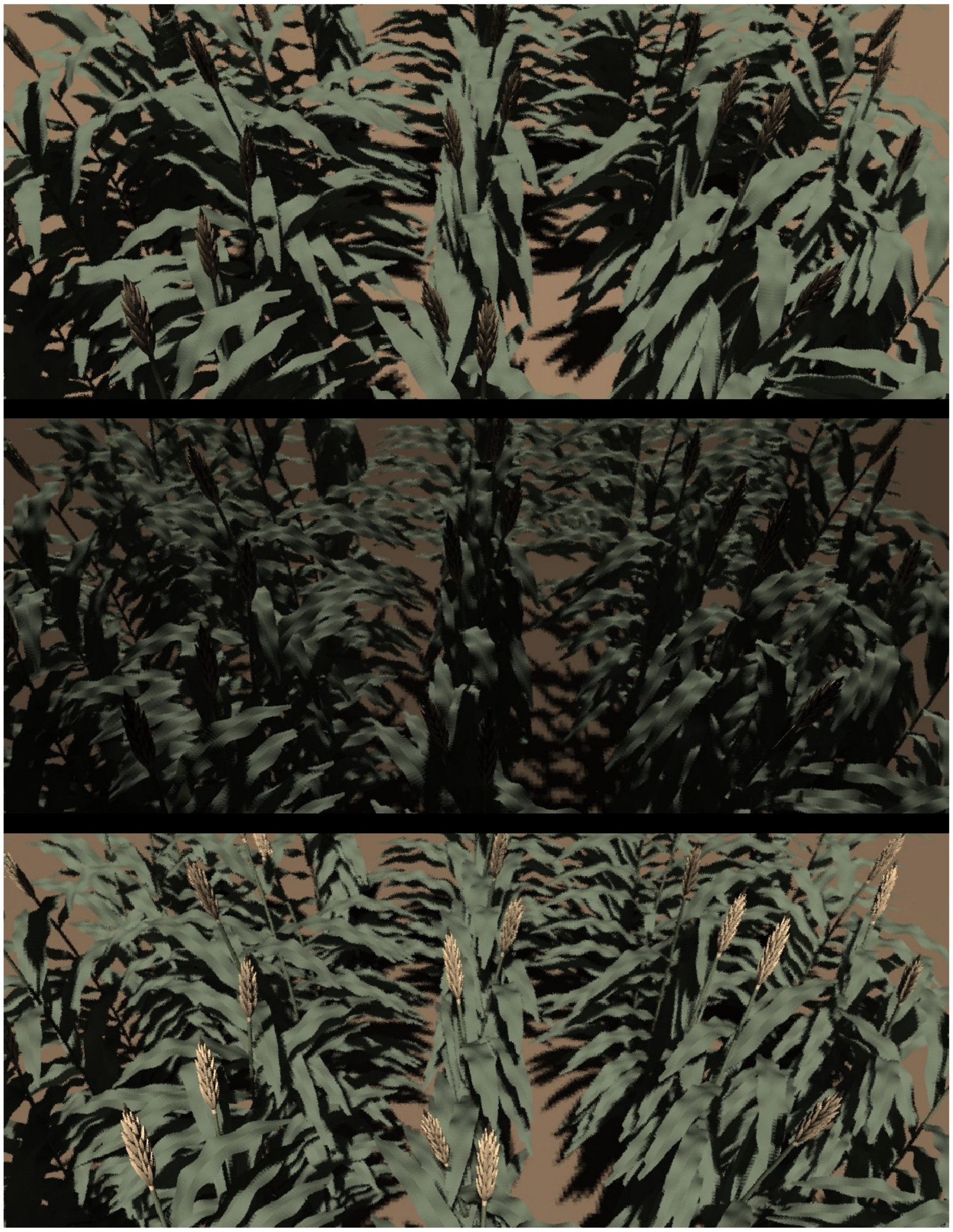

Figure S8: Synthetic image of sorghum plants under direct sunlight with various zenith (0°, 60°, 30°from top to bottom) and azimuth (0°, 90°, 270°from top to bottom) angles. The source flux varies with the zenith angle, while the scene and the simulated camera remain constant. The camera focal plane distance, HFOV, and diameter of lens are 1.35 m, 60°, and 0.02 m, respectively

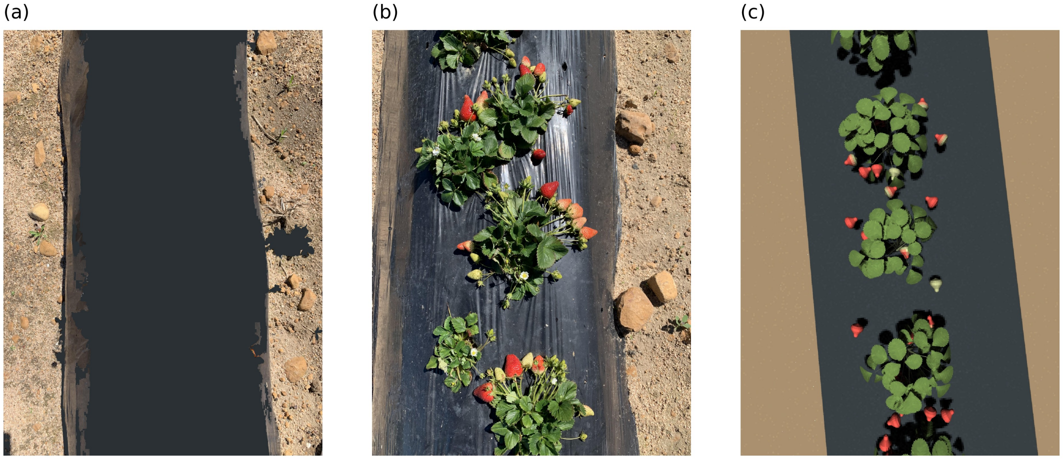

Figure S9: Example images from strawberry detection case study. (a) Background image, (b) Real strawberry RGB image; (c) Synthetic strawberry RGB image.

Figure S7: Schematic depiction of depth image calculation. The red point is the target primitive.

Figure S8: Synthetic image of sorghum plants under various sunlight conditions.

Figure S9: Background, real and synthetic images of strawberry plants.

Supplementary 6 Thermal and depth imaging

Simulated thermal image generation is also an integral part of our framework. The thermal image represents the surface temperature (Kelvin) of objects in the scene, calculated using the energy balance equation, which incorporates emissivity and the Stefan-Boltzmann law [58]. A key parameter in this equation is the longwave radiation flux, and it can be obtained through ray-tracing. The present radiation model initially computes this flux on each primitives, then it is coupled with the Helios Energy Balance Model plug-in to generate thermal images. Other parameters, like leaf conductance to moisture, can be calculated using Stomatal Conductance Model plug-in [25].

Pixels can also be labeled based on the distance from the camera to the nearest object to produce a "depth" image, similar to what might be generated via a stereo camera [59] or a structure from motion algorithm [60]. The depth of a pixel is determined by measuring the distance between the lens plane and the object plane rather than the distance from the lens center to the object point (Fig. S7), which is consistent with typical real depth cameras. The object plane, which houses the target primitive, possesses a normal that stands perpendicular to the lens plane.
