# Supplementary figures and images for: Simulation of Automatically Annotated Visible and Multi-/Hyperspectral Images Using the Helios 3D Plant and Radiative Transfer Modeling Framework
Source: Plant Phenomics. 2024 May 30;6:0189. doi: 10.34133/plantphenomics.0189 (PMC11136674; doi:10.34133/plantphenomics.0189)

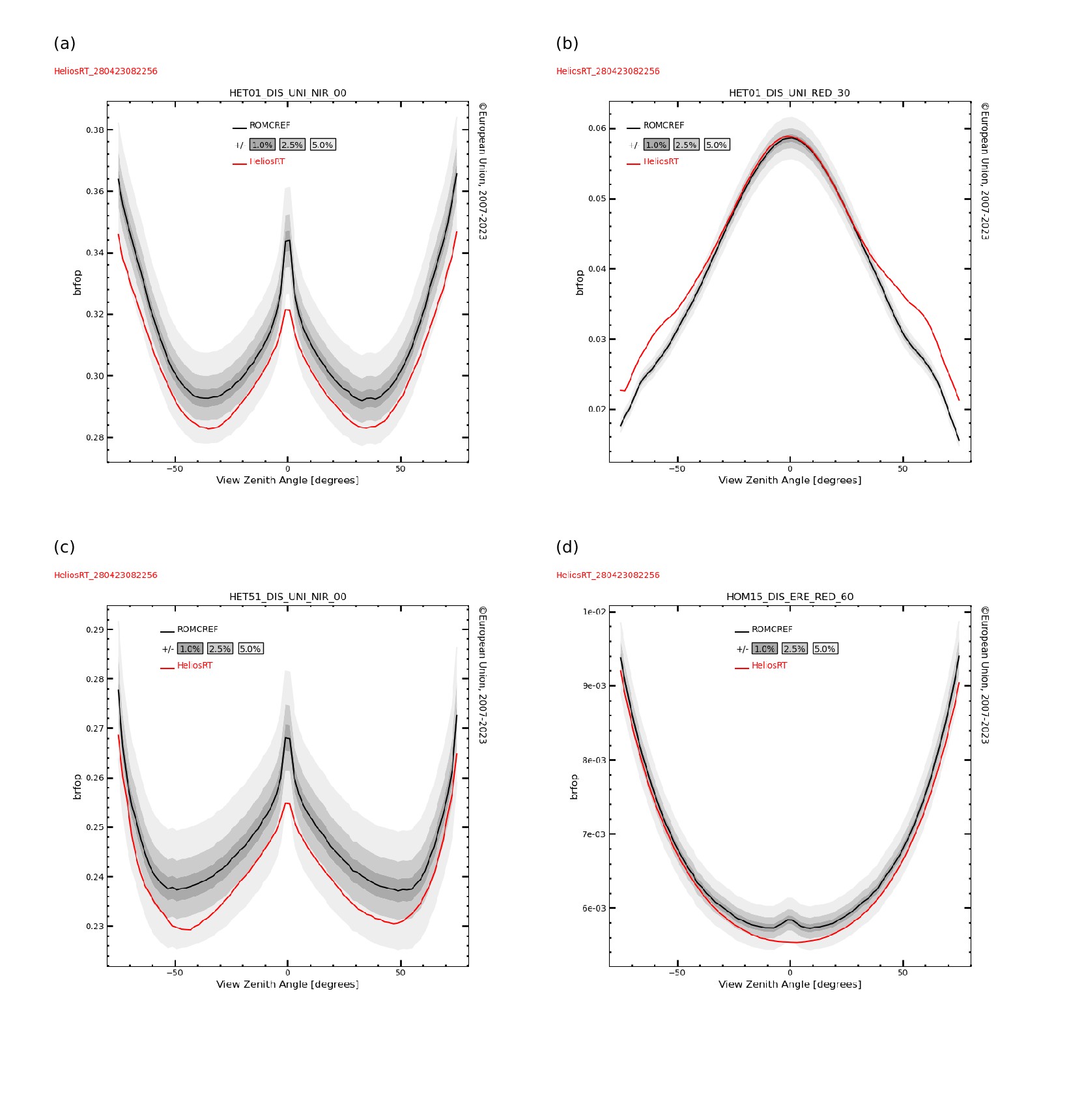

Supplement: Supplementary 1 — Sections S1 to S6 Figs. S1 to S9 Tables S1 and S2 References [54–60] [file plantphenomics.0189.f1.zip › figure_s1.jpeg]

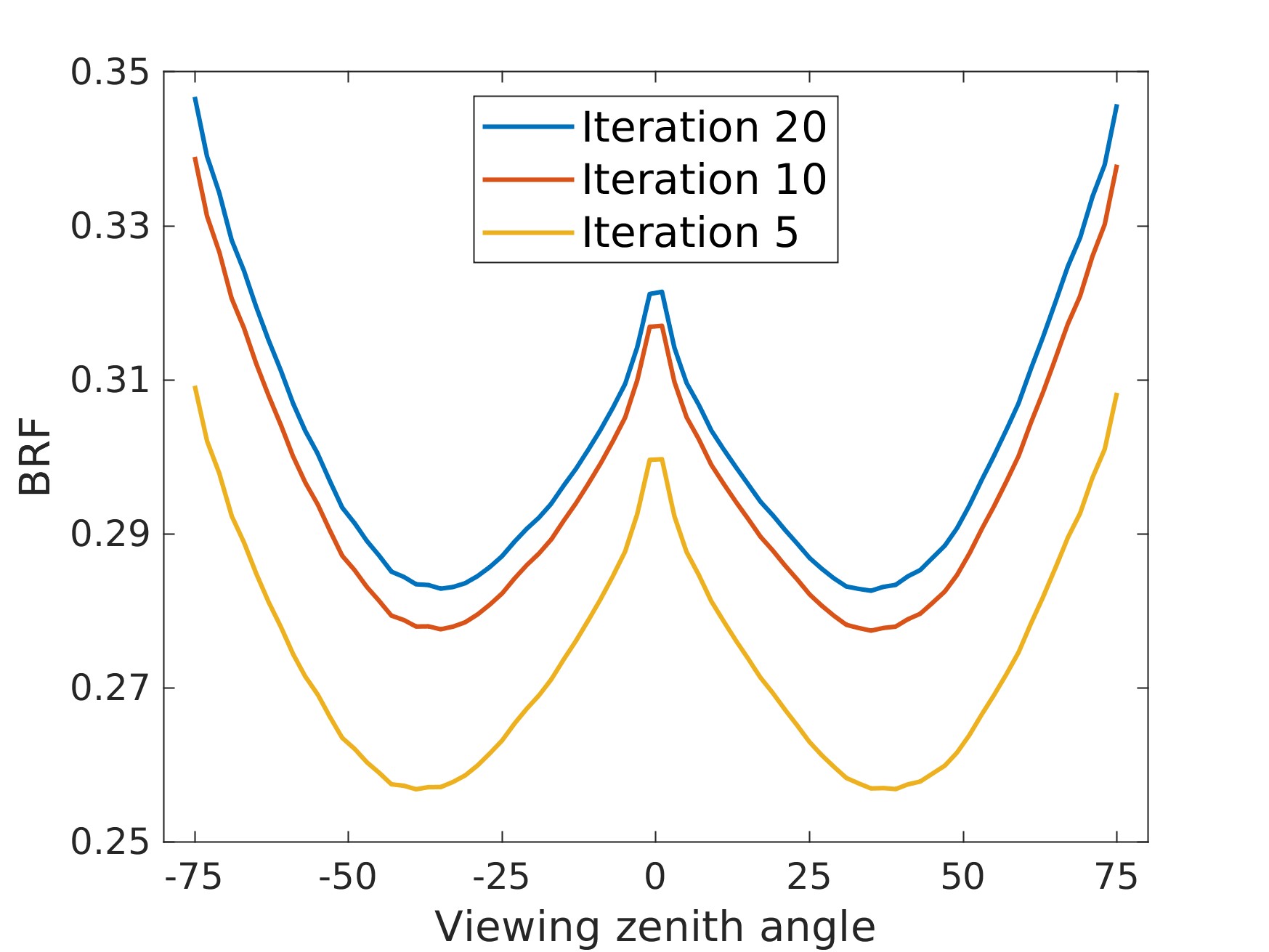

Supplement: Supplementary 1 — Sections S1 to S6 Figs. S1 to S9 Tables S1 and S2 References [54–60] [file plantphenomics.0189.f1.zip › figure_s2.jpeg]

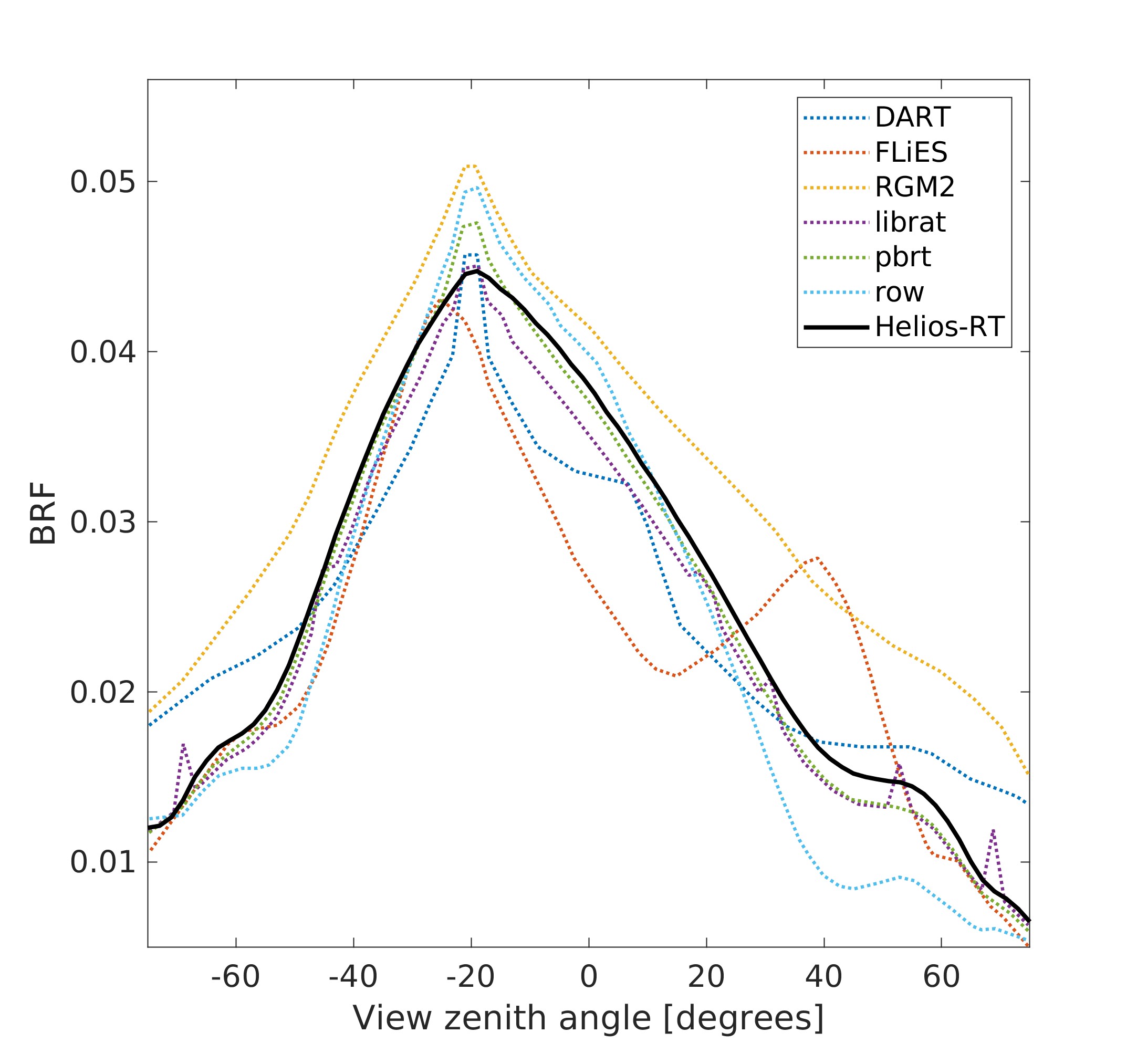

Supplement: Supplementary 1 — Sections S1 to S6 Figs. S1 to S9 Tables S1 and S2 References [54–60] [file plantphenomics.0189.f1.zip › figure_s3.jpeg]

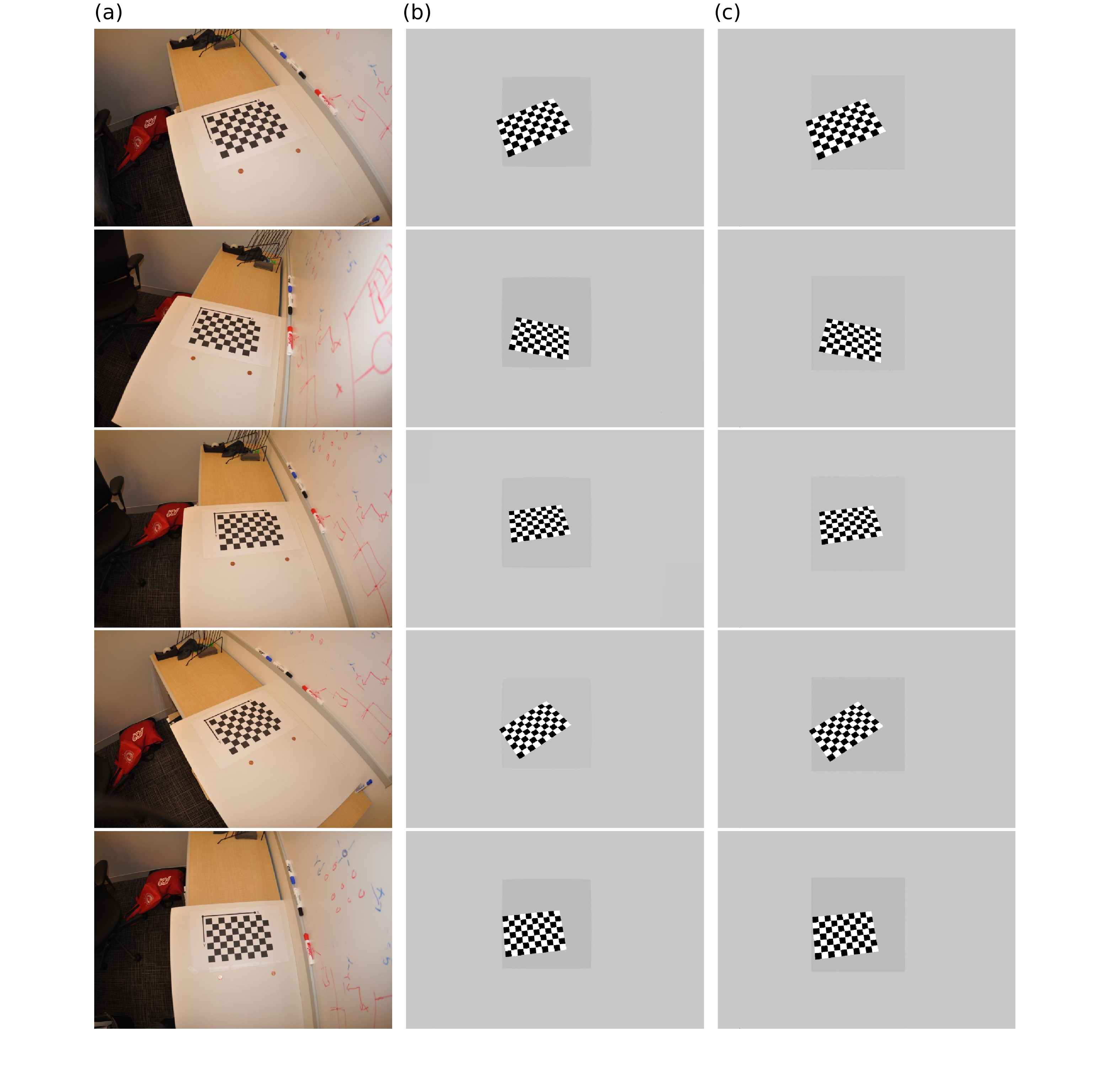

Supplement: Supplementary 1 — Sections S1 to S6 Figs. S1 to S9 Tables S1 and S2 References [54–60] [file plantphenomics.0189.f1.zip › figure_s4.jpeg]

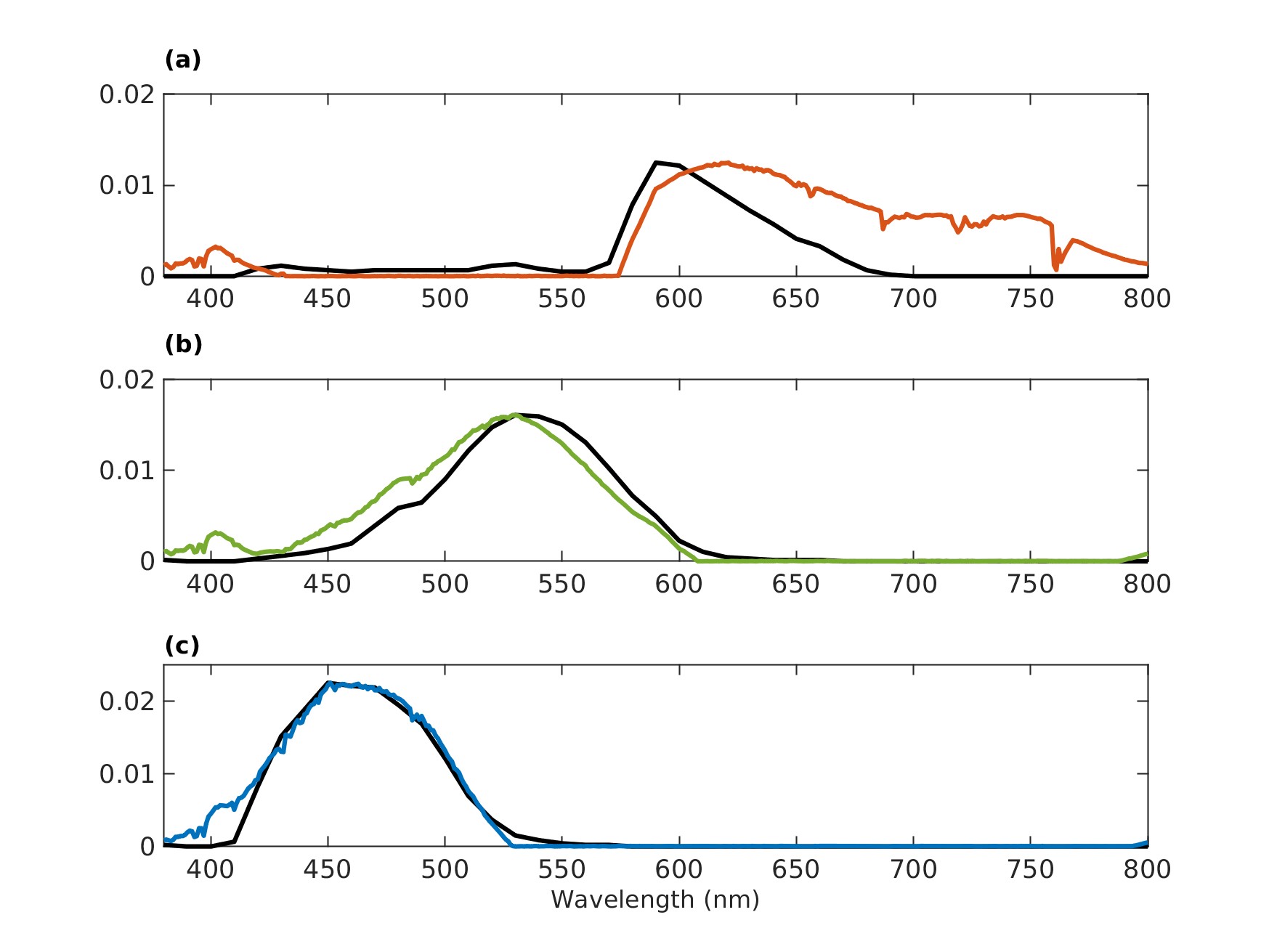

Supplement: Supplementary 1 — Sections S1 to S6 Figs. S1 to S9 Tables S1 and S2 References [54–60] [file plantphenomics.0189.f1.zip › figure_s5.jpeg]

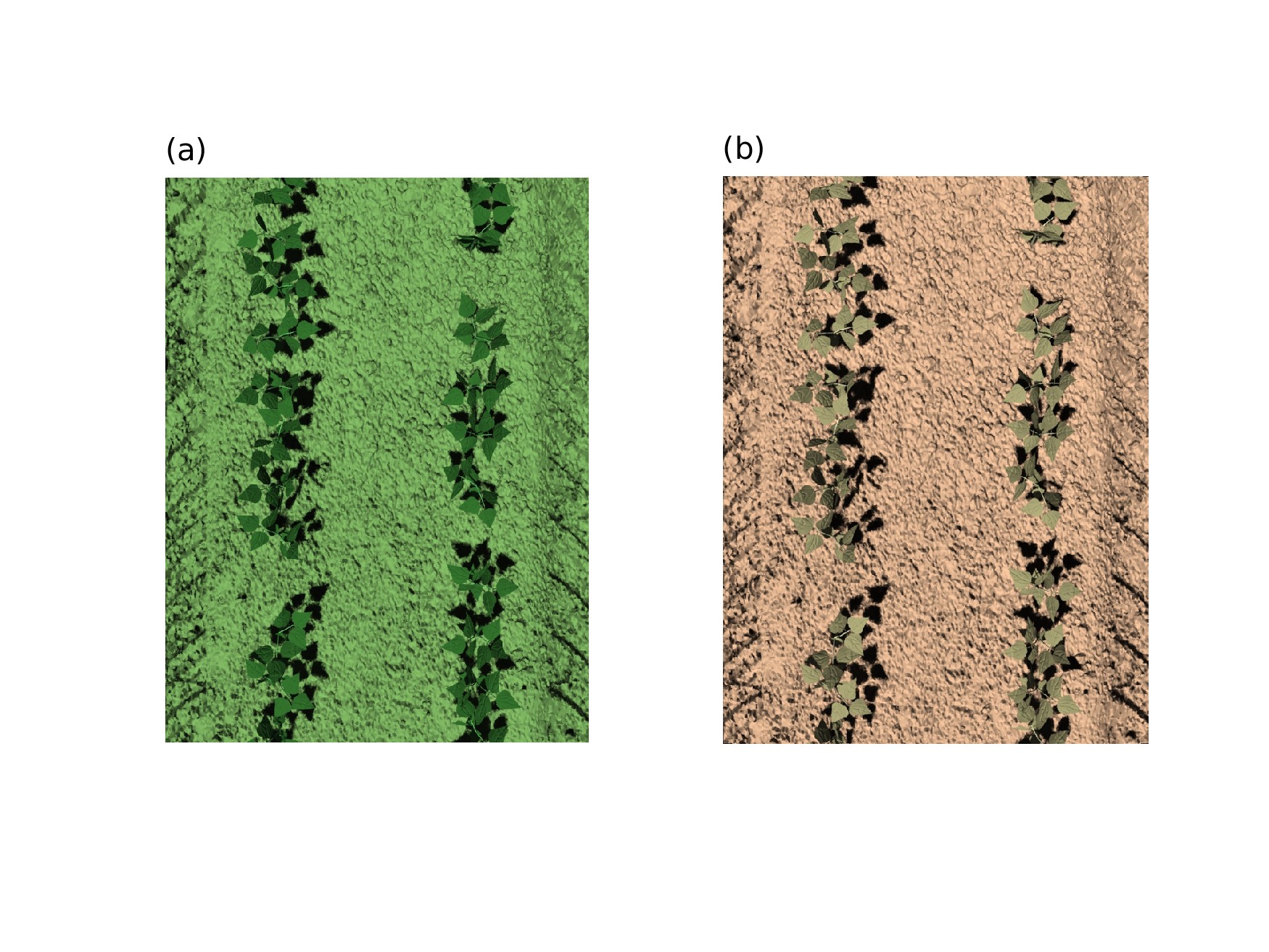

Supplement: Supplementary 1 — Sections S1 to S6 Figs. S1 to S9 Tables S1 and S2 References [54–60] [file plantphenomics.0189.f1.zip › figure_s6.jpeg]

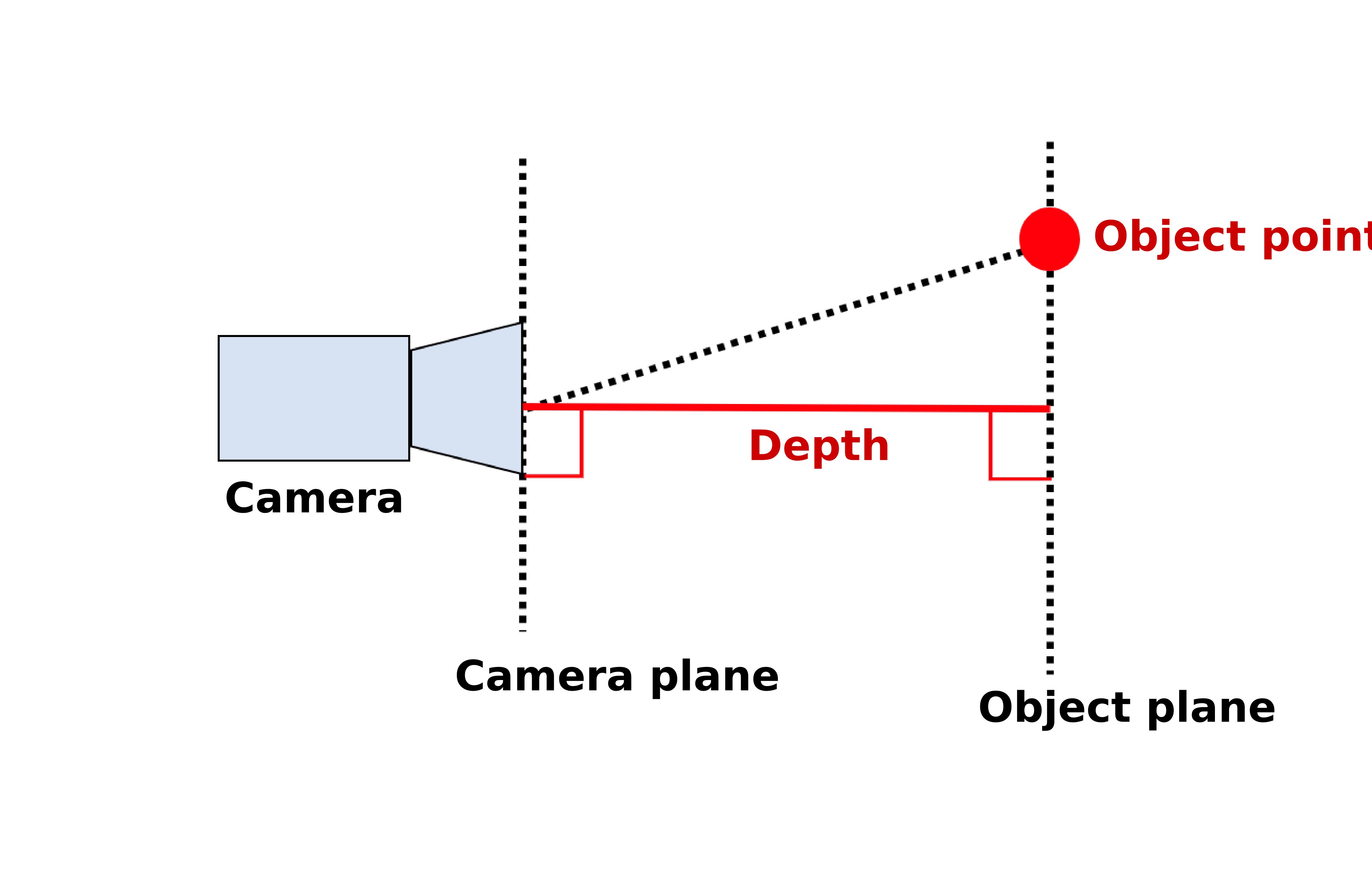

Supplement: Supplementary 1 — Sections S1 to S6 Figs. S1 to S9 Tables S1 and S2 References [54–60] [file plantphenomics.0189.f1.zip › figure_s7.jpeg]

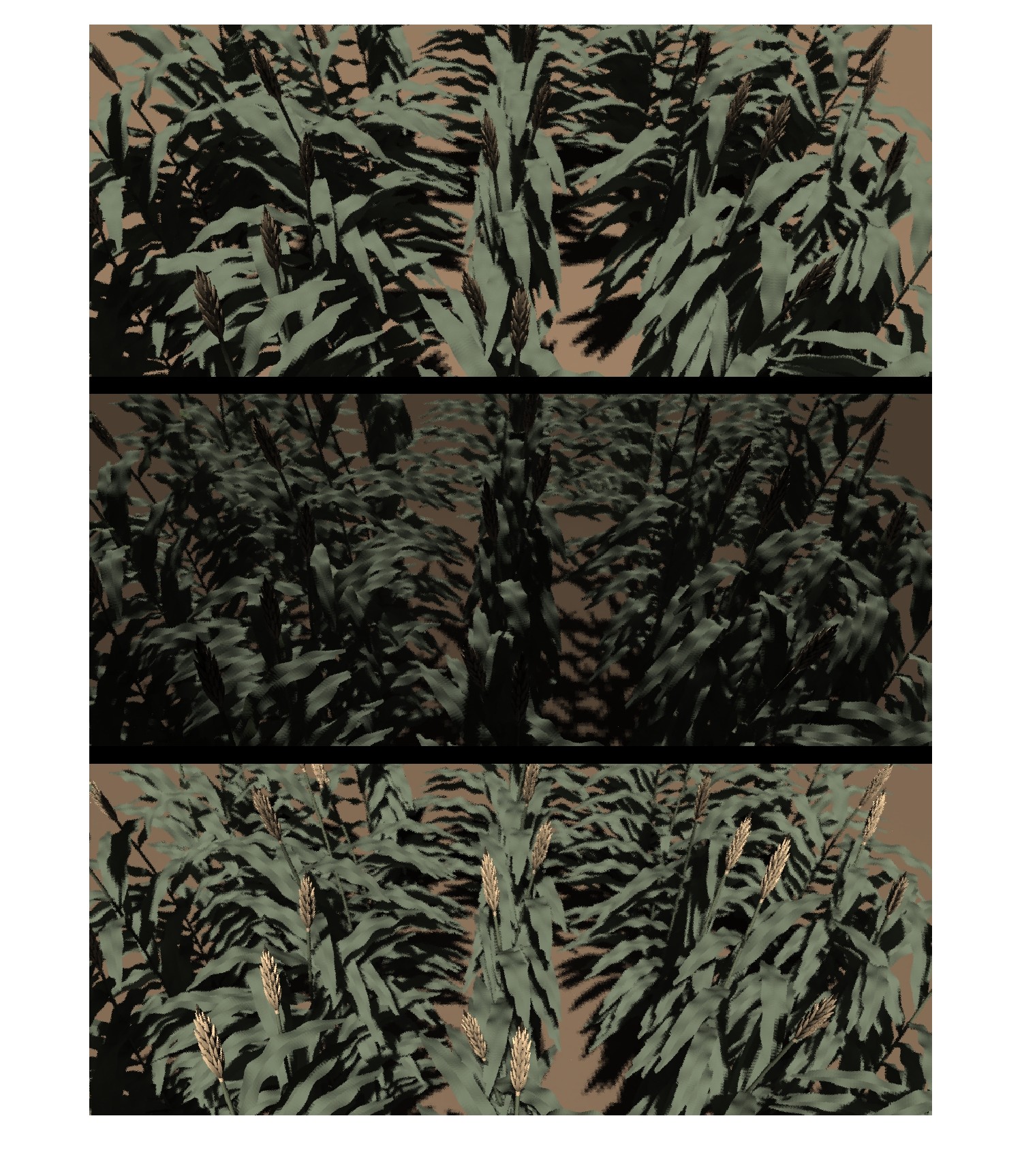

Supplement: Supplementary 1 — Sections S1 to S6 Figs. S1 to S9 Tables S1 and S2 References [54–60] [file plantphenomics.0189.f1.zip › figure_s8.jpeg]

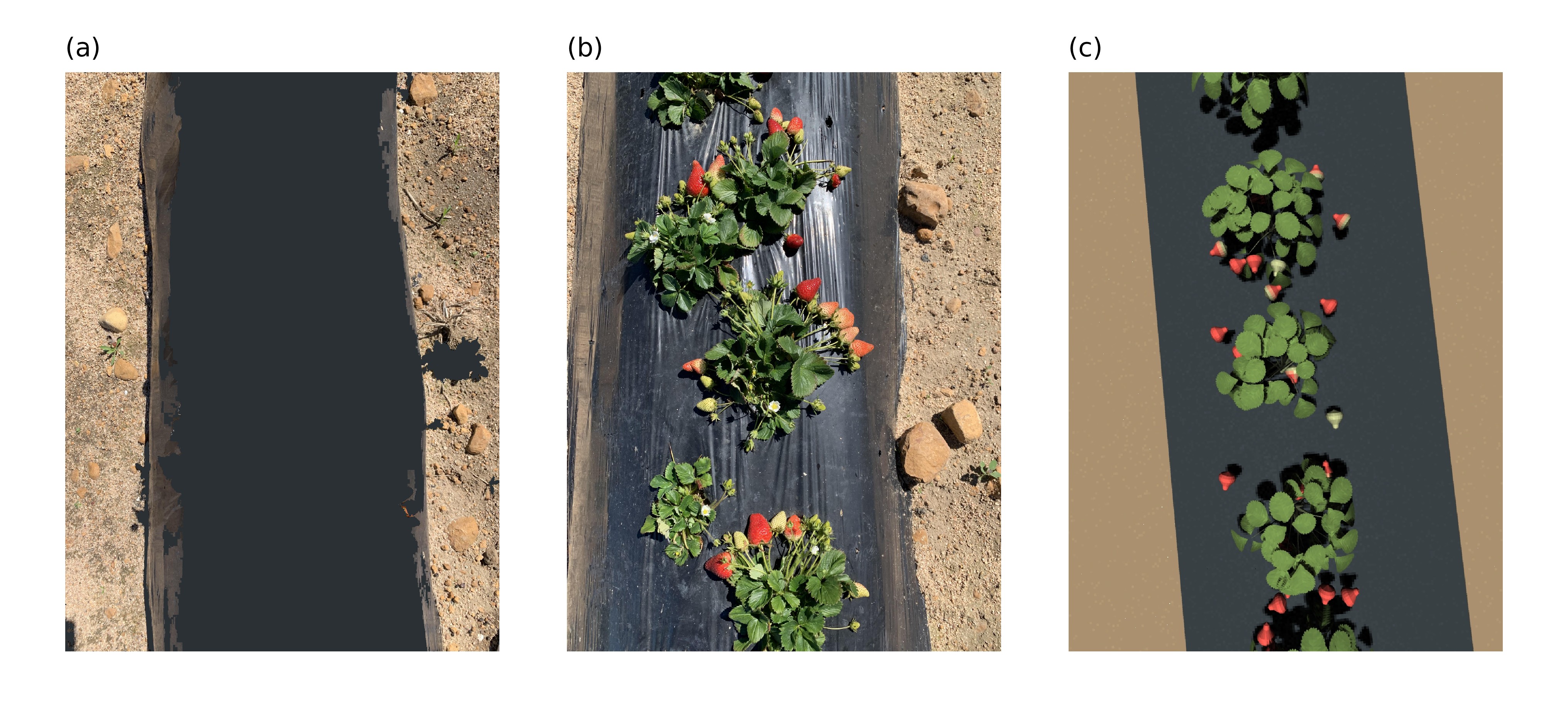

Supplement: Supplementary 1 — Sections S1 to S6 Figs. S1 to S9 Tables S1 and S2 References [54–60] [file plantphenomics.0189.f1.zip › figure_s9.jpg]
